# Identification of SaCas9 orthologs containing a conserved serine residue that determines simple NNGG PAM recognition

**DOI:** 10.1371/journal.pbio.3001897

**Published:** 2022-11-30

**Authors:** Shuai Wang, Chen Tao, Huilin Mao, Linghui Hou, Yao Wang, Tao Qi, Yuan Yang, Sang-Ging Ong, Shijun Hu, Renjie Chai, Yongming Wang

**Affiliations:** 1 State Key Laboratory of Genetic Engineering, School of Life Sciences, Zhongshan Hospital, Fudan University, Shanghai, China; 2 Department of Pharmacology, University of Illinois College of Medicine, Chicago, Illinois, United States of America; 3 Division of Cardiology, Department of Medicine, University of Illinois College of Medicine, Chicago, Illinois, United States of America; 4 Department of Cardiovascular Surgery of the First Affiliated Hospital & Institute for Cardiovascular Science, Collaborative Innovation Center of Hematology, State Key Laboratory of Radiation Medicine and Protection, Suzhou Medical College, Soochow University, Suzhou, China; 5 State Key Laboratory of Bioelectronics, Department of Otolaryngology Head and Neck Surgery, Zhongda Hospital, School of Life Sciences and Technology, Advanced Institute for Life and Health, Jiangsu Province High-Tech Key Laboratory for Bio-Medical Research, Southeast University, Nanjing, China; 6 Co-Innovation Center of Neuroregeneration, Nantong University, Nantong, China; 7 Department of Otolaryngology Head and Neck Surgery, Sichuan Provincial People’s Hospital, University of Electronic Science and Technology of China, Chengdu, China; 8 Shanghai Engineering Research Center of Industrial Microorganisms, Shanghai, China; ETH Zurich, SWITZERLAND

## Abstract

Due to different nucleotide preferences at target sites, no single Cas9 is capable of editing all sequences. Thus, this highlights the need to establish a Cas9 repertoire covering all sequences for efficient genome editing. Cas9s with simple protospacer adjacent motif (PAM) requirements are particularly attractive to allow for a wide range of genome editing, but identification of such Cas9s from thousands of Cas9s in the public database is a challenge. We previously identified PAMs for 16 SaCas9 orthologs. Here, we compared the PAM-interacting (PI) domains in these orthologs and found that the serine residue corresponding to SaCas9 N986 was associated with the simple NNGG PAM requirement. Based on this discovery, we identified five additional SaCas9 orthologs that recognize the NNGG PAM. We further identified three amino acids that determined the NNGG PAM requirement of SaCas9. Finally, we engineered Sha2Cas9 and SpeCas9 to generate high-fidelity versions of Cas9s. Importantly, these natural and engineered Cas9s displayed high activities and distinct nucleotide preferences. Our study offers a new perspective to identify SaCas9 orthologs with NNGG PAM requirements, expanding the Cas9 repertoire.

## Abstract 2

Due to different nucleotide preferences at target sites, no single Cas9 is capable of editing all sequences. This study identifies variants in the PAM-interacting domain of natural SaCas9 orthologs that recognize a simple NNGG PAM, and demonstrates that introducing these variants can confer NNGG PAM recognition, expanding the Cas9 repertoire.

## Introduction

The Clustered Regularly Interspaced Short Palindromic Repeats (CRISPR)-RNA-guided Cas endonuclease system is based on the bacterial adaptive immune system and has been utilized as a fast and efficient method for precise genome editing [1–6]. This system is made up of two main components: a Cas9 nuclease and a chimeric single-guide RNA (sgRNA) derived from CRISPR RNA (crRNA) and the *trans*-activating crRNA (tracrRNA) [2]. Cas9 and sgRNA combine to form a complex that recognizes the target DNA that is complementary to the 5′ end of the sgRNA [2]. In addition to sgRNA-target DNA complementarity, DNA recognition requires a specific DNA sequence known as protospacer adjacent motif (PAM), flanking the target sequence [2]. The PAM allows the Cas nuclease to discriminate between the target DNA and the DNA sequence encoding the sgRNA but also restricts its ability to target any sequence in the genome.

Editing efficiency is a major hurdle of the CRISPR system. Every Cas nuclease has its own nucleotide preference [7]. For example, SpCas9 prefers guanine-rich sequences [8], while AsCas12a prefers adenine-rich sequences [9]. SpCas9 is generally considered the most efficient Cas nuclease, whose efficiency varies from 0% to approximately 100% depending on the target sequences [8]. Although previous studies have focused on limitations of the PAM [10–12], the sole presence of a PAM within a locus does not guarantee that it can be efficiently edited. For high efficiency of genome editing to be achieved, it is essential to establish a Cas9 repertoire that can accommodate all sequences.

Cas9 nucleases with flexible PAM requirements are crucial for large-scale genome editing. We previously developed Cas9 nucleases with highly flexible NNGG PAMs recognition [13,14]. To rapidly identify additional natural Cas9 nucleases recognizing NNGG PAMs, we compared the PAM-interacting (PI) domains of SaCas9 orthologs and found that the serine residue corresponding to SaCas9 N986 was associated with the NNGG PAM. We identified five additional SaCas9 orthologs recognizing the NNGG PAM. We further engineered two of them to improve the specificity. Our study expands the Cas9 repertoire and provides a foundation to search for Cas9s with NNGG PAMs in the future.

## Results

### A serine residue was associated with the NNGG PAM requirement among SaCas9 orthologs

**Fig 1:**
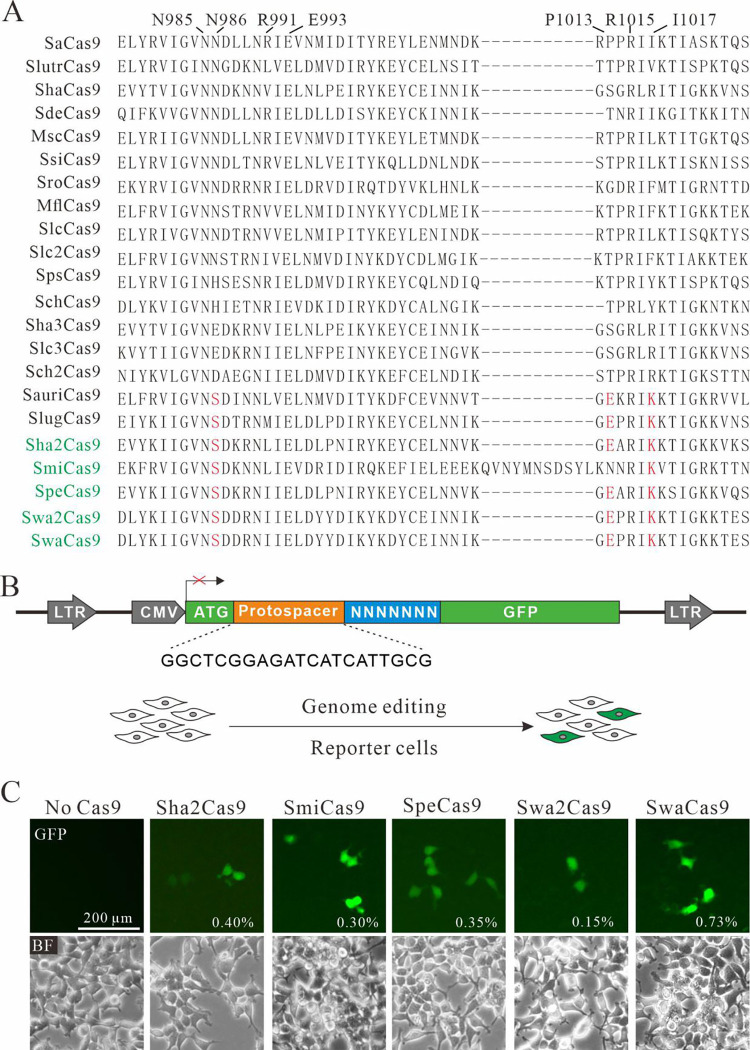
Analysis of five SaCas9 ortholog activities. (**A**) Amino acid sequences of the SaCas9 ortholog PI domain are aligned. The residues that are important for PAM recognition are indicated at the top; the conserved residues among newly identified SaCas9 orthologs are shown in red; the names of newly identified Cas9s are shown in green. (**B**) Design of the GFP activation reporter construct. A target sequence (protospacer) containing a 7-bp random sequence is inserted between ATG and the GFP-coding sequence. The library DNA is stably integrated into HEK293T cells by lentivirus. (**C**) Transfection of SaCas9 orthologs induced GFP expression. Percentage of GFP-positive cells was shown. The cells without transfection of Cas9 were used as a negative control.

We previously identified PAMs for 16 SaCas9 orthologs, where SauriCas9 and SlugCas9 recognized NNGG PAMs [13–15]. Nishimasu and colleagues have demonstrated that amino acids of N985, N986, R991, E993, and R1015 in the PI domain of SaCas9 are crucial for PAM recognition [16]. N985, E993, and R1015 are very conserved among these 16 orthologs (Fig 1A). In contrast, residues corresponding to N986 and R991 showed substantial diversity. Interestingly, SauriCas9 and SlugCas9 contain a serine residue corresponding to SaCas9 N986. We hypothesized that this serine residue is associated with the NNGG PAM.

We employed SaCas9 as a template to search for related orthologs from NCBI’s Gene database, and our search identified five additional Cas9s that contained this serine residue with amino acid identity ranging from 58.4% to 64.5% (Fig 1A and Table 1). Genetic loci of these orthologs contain a conserved organization where Cas9 is followed by Cas1 and Cas2 (S1A Fig). The organization of CRISPR repeats and tracrRNAs does not appear to be conserved. SaCas9 encodes a tracrRNA upstream of Cas9 and CRISPR repeats downstream of Cas2. Sha2Cas9 and SpeCas9 encode CRISPR repeats and tracrRNAs downstream of Cas2. SwaCas9 and Swa2Cas9 encode CRISPR repeats and tracrRNAs upstream of Cas9 and additional CRISPR repeats downstream of Cas2. SmiCas9 encodes CRISPR repeats-tracrRNA-CRISPR repeats upstream of Cas9.

**Table 1:** Five SaCas9 orthologs selected from the NCBI database.

NCBI ID	Host strain	Name	Length (aa)	Identity to SaCas9
Sha2Cas9	*Staphylococcus haemolyticus*	WP_154836552	1,058	63.2%
SmiCas9	*Staphylococcus microti*	WP_044361501	1,063	58.4%
SpeCas9	Staphylococcus petrasii	WP_115359133	1,058	63.5%
SwaCas9	*Staphylococcus warneri*	WP_107532850	1,054	64.5%
Swa2Cas9	*Staphylococcus warneri*	WP_114599540	1,054	64.3%

Nevertheless, the 5′ end sequences of CRISPR repeats and tracrRNAs exhibited high conservation among these orthologs (S1B and S1C Fig). We fused the 3′ end of a direct repeat with the 5′ end of the corresponding tracrRNA, including the full-length tail, via a 4-nt linker to form a sgRNA for each Cas9 (S2A Fig). Interestingly, these sgRNAs formed a similar secondary structures with three stem loops (S2B Fig), suggesting that these SaCas9 orthologs could share the same sgRNA scaffold for genome editing.

### PAM screening

**Fig 2:**
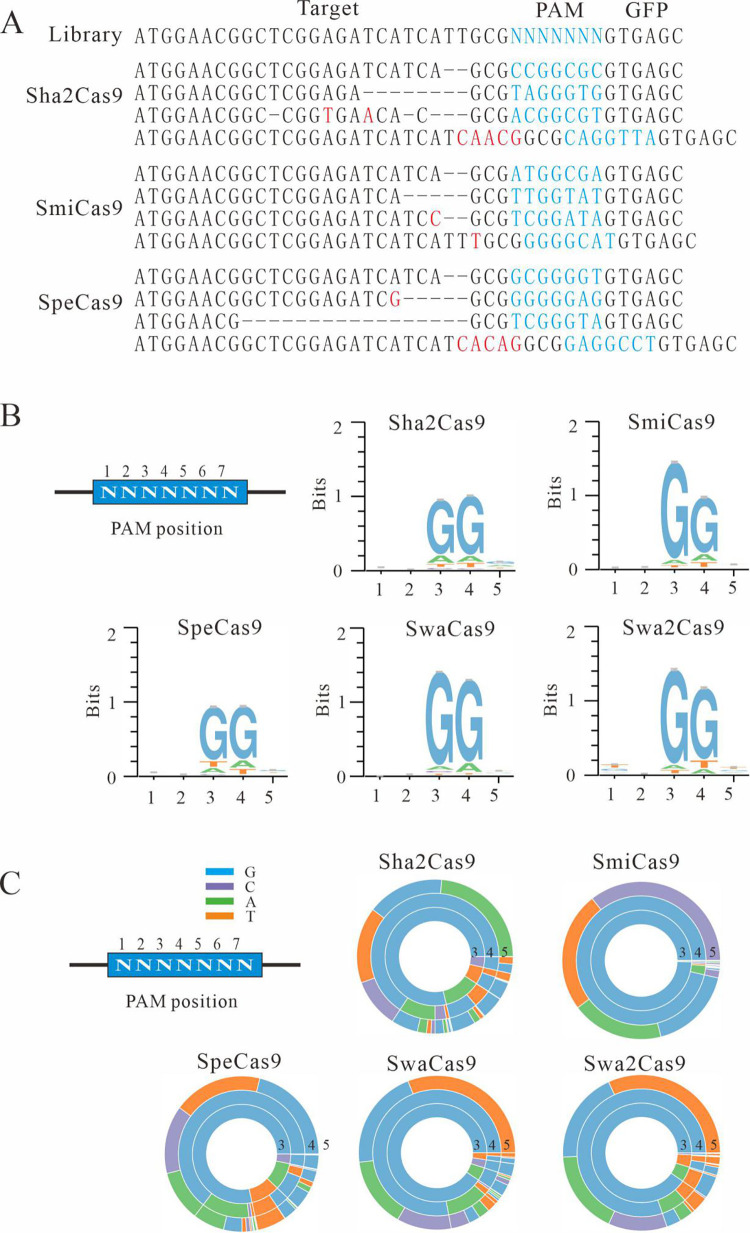
Analysis of the PAM sequence of Cas9. (**A**) Deep sequencing reveals that SmiCas9, Sha2Cas9, and SpeCas9 generated indels on the targets. (**B**) WebLogos were generated based on the deep sequencing data. (**C**) PAM wheels were generated based on the deep sequencing data.

Next, we used a previously established GFP activation assay for PAM screening [13,15]. In this assay, the GFP expression is disrupted by a target sequence (protospacer) flanked by a 7-bp random sequence, which is inserted into the GFP coding sequence immediately downstream of the ATG start codon, inducing to a frameshift mutation. This reporter library is then stably integrated into HEK293T cells. If a Cas9 nuclease successfully edits the target sequence, small insertions or deletions (indels) will be generated at the target sequence, and a functional GFP cassette will be restored in a portion of cells (Fig 1B). Each Cas9 was human codon optimized, synthesized, and cloned into a mammalian expression construct that was developed by Ran and colleagues [17]. The canonical SaCas9 sgRNA scaffold was employed for sgRNA expression [17]. Three days after transfection of Cas9 with sgRNA expression plasmids, all five tested Cas9s induced GFP expression (Fig 1C). GFP-positive cells were sorted out and the target DNA was PCR amplified for deep sequencing. Sequencing results showed that indels occurred at target sites (Fig 2A). WebLogos and PAM wheels were generated based on deep sequencing data, which revealed that these Cas9s recognized NNGG PAMs (Fig 2B and 2C). These data validated our hypothesis that the serine residue is associated with the NNGG PAM.

To test whether the serine residue corresponding to SaCas9 N986 determined NNGG PAM recognition, we replaced N986 with a serine (S3A Fig). The GFP activation assay revealed that the substitution increased guanine preference at PAM position 3, but the favored PAM remained NNGRRT (S3B Fig). We reanalyzed the PI domain of these orthologs and identified two additional residues, a glutamic acid (E) corresponding to SaCas9 P1013 and a lysine (K) corresponding to SaCas9 I1017, conserved among orthologs that recognized an NNGG PAM, except SmiCas9 where there is a 13-amino acid insertion corresponding to SaCas9 P1013 (Fig 1A). We added either P1013E, I1017K, or both mutations to SaCas9-N986 (S3A Fig). Interestingly, all resulted SaCas9 variants recognized an NNGG PAM (S3B Fig). These data demonstrated that these three amino acids are important for determining the NNGG PAM.

### Genome editing for endogenous loci

Next, we tested the capacity of these Cas9s for genome editing at selected endogenous sites in HEK293T cells. Five days after transfection of Cas9 and sgRNA expression plasmid DNA, we extracted genomic DNA and amplified target sites by PCR. As an initial screen, we used the T7EI assay to rapidly analyze the efficiency for each Cas9. SmiCas9, Sha2Cas9, and SpeCas9 displayed higher editing efficiency, while SwaCas9 and Swa2Cas9 displayed lower editing efficiency (S4A and S4B Fig). In the subsequent experiments, we only focused on SmiCas9, Sha2Cas9, and SpeCas9.

**Fig 3:**
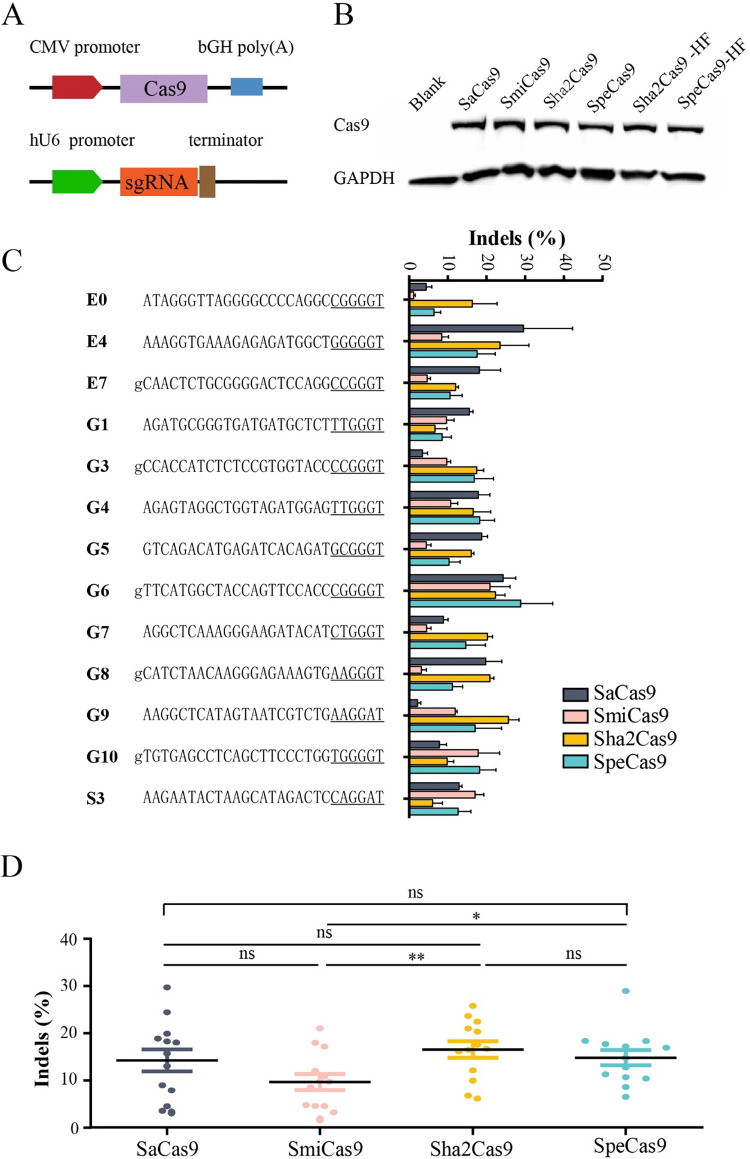
Genome editing for endogenous sites. (**A**) Schematic of the Cas9 expression constructs. (**B**) Protein expression level of Cas9s was measured by western blot. Cells without Cas9 transfection was used as a negative control. (**C**) Comparison of SaCas9, SmiCas9, Sha2Cas9, and SpeCas9 efficiency for genome editing at 13 endogenous loci. Additional “g” is added for U6 promoter transcription (*n =* 3). Underlying data for all summary statistics can be found in S1 Data. (**D**) Quantification of editing efficiency for SaCas9, SmiCas9, Sha2Cas9, and SpeCas9. Underlying data for all summary statistics can be found in S1 Data.

We compared the activity of these three Cas9s to that of SaCas9 at 13 endogenous sites with NNGGRT PAMs. All tested Cas9s were expressed from the same construct and achieved similar expression levels, as revealed by western blot (Fig 3A and 3B). All four Cas9 nucleases generated indels with different efficiencies depending on the target sites in HEK293T cells (Fig 3C). Interestingly, these Cas9s displayed different activities at some sites. For example, Sha2Cas9 displayed higher activity at site E0, while SmiCas9 and SpeCas9 displayed higher activity at site G10. SaCas9 displayed lower efficiency than newly identified Cas9s at sites G3 and G9. These data demonstrated that these Cas9s prefer distinct target sequences. Overall, SaCas9, Sha2Cas9, and SpeCas9 displayed comparable activities, while SmiCas9 displayed lower activity (Fig 3D).

### Specificity of SmiCas9, Sha2Cas9, and SpeCas9

Next, we compared the specificity of SmiCas9, Sha2Cas9, SpeCas9, and SaCas9 using the GFP activation assay. A panel of sgRNAs with dinucleotide mutations along the protospacer was generated to detect the specificity of each Cas9. Off-target cleavage is considered to have occurred when the mismatched sgRNAs induce GFP expression. Overall, SaCas9 and SmiCas9 had negligible off-target effects, while Sha2Cas9 and SpeCas9 displayed moderate off-target effects (S5 Fig). Specifically, SaCas9 was highly sensitive to mismatches at PAM-proximal and PAM-distal positions but relatively less sensitive at middle positions; SmiCas9 displayed minimal off-target effects with mismatches at all positions; and Sha2Cas9 and SpeCas9 were sensitive to mismatches at PMA-proximal positions 18 through 20 but less sensitive at other positions.

**Fig 4:**
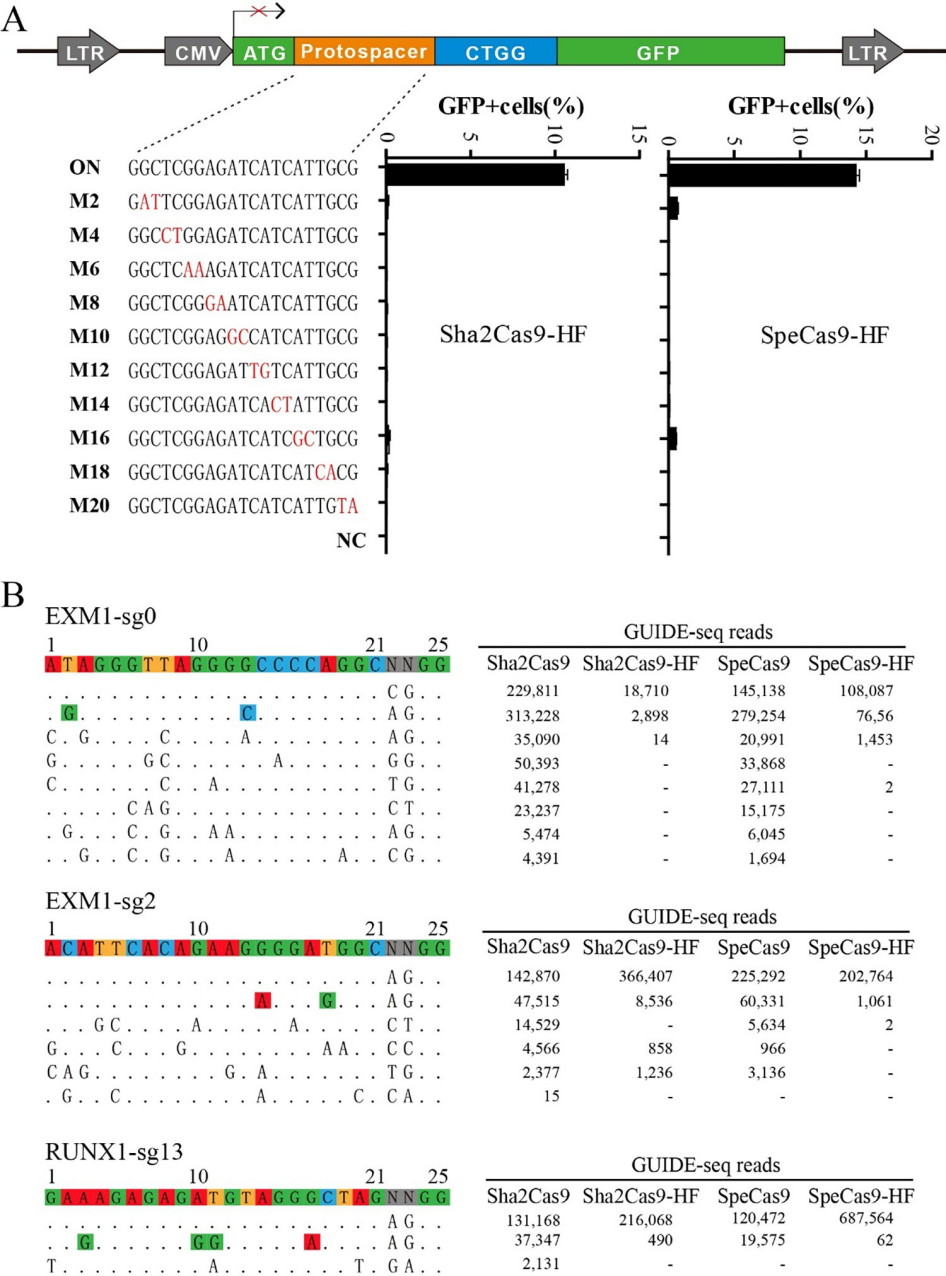
Analysis of Sha2Cas9-HF and SpeCas9-HF specificity. (**A**) Schematic of the GFP activation assay for specificity analysis is shown on the top. A panel of sgRNAs with dinucleotide mutations is shown below. sgRNA activities were measured based on GFP expression. Mismatches are shown in red (*n =* 3). Underlying data for all summary statistics can be found in S1 Data. (**B**) Off-targets for EMX1 locus are analyzed by GUIDE-seq. Read numbers for on- and off-targets are shown on the right. Mismatches compared with the on-target site are shown and highlighted in color.

Recently, Tan and colleagues unraveled the crystal structure of the SaCas9/sgRNA–target DNA complex and identified four amino acid residues (R245, N413, N419, and R654) forming polar contacts within a 3.0-Å distance from the target DNA strand [18]. When one or more of these residues were replaced by alanine, SaCas9 specificity was significantly improved [18]. To investigate whether the specificity of Sha2Cas9 can be improved, we used pairwise alignment to identify the corresponding residues (R247, N415, S421, and R656; S6 Fig) and generated single amino acid mutants by alanine substitution. The GFP activation assay revealed that the R247A and N415A mutations could significantly improve specificity without compromising the on-target activity (S7A and S7B Fig). The R656A mutation also improved the specificity although this was accompanied by markedly decreased on-target activity. We introduced the R247A and N415A double mutations into Sha2Cas9 to generate a high-fidelity version of Cas9 named Sha2Cas9-HF. The GFP activation assay revealed that double mutations further improved its specificity (Fig 4A).

We simultaneously identified the corresponding residues for SpeCas9 (R247, N415, S421, and R656; S6 Fig) and generated single amino acid mutants by alanine substitution (S8A Fig). The GFP activation assay revealed that the R247A, N415A, and S421A mutations could significantly improve specificity without compromising the on-target activity (S8B Fig). We introduced the R247A, N415A, and S421A triple mutations into SpeCas9 to generate a high-fidelity version of Cas9 named SpeCas9-HF. The GFP activation assay revealed that triple mutations further improved specificity (Fig 4A).

Genome-wide unbiased off-target effects of Sha2Cas9, Sha2Cas9-HF, SpeCas9, and SpeCas9-HF were next evaluated by GUIDE-seq [19]. We evaluated two sites targeting the EXM1 gene and one site targeting the RUNX1 gene. Five days after transfection of the Cas9 plasmid, the sgRNA plasmid, and the GUIDE-seq oligos, we prepared libraries for deep sequencing. Sequencing and analysis showed that on-target cleavage occurred for all Cas9 nucleases at 3 targets, as reflected by the high GUIDE-seq read counts (Fig 4B). High-fidelity versions of Cas9s displayed significantly fewer off-target effects than wild-type Cas9s, reflected by the numbers of off-target sites and off-target read counts. For example, SpeCas9 and SpeCas9-HF generated similar read counts (225,292 versus 202,764) at the EXM1-sg2 site. SpeCas9 induced four off-target sites, while SpeCas9-HF induced two off-target sites. For one off-target, SpeCas9 generated 60,331 read counts, while SpeCas9-HF generated 1,061 read counts. For another off-target, SpeCas9 generated 5,634 read counts, while SpeCas9-HF generated 2 read counts. These data demonstrated that the occurrence of off-target events is significantly lower when using Sha2Cas9-HF and SpeCas9-HF.

### Evaluation of Sha2Cas9-HF and SpeCas9-HF on-target activities

**Fig 5:**
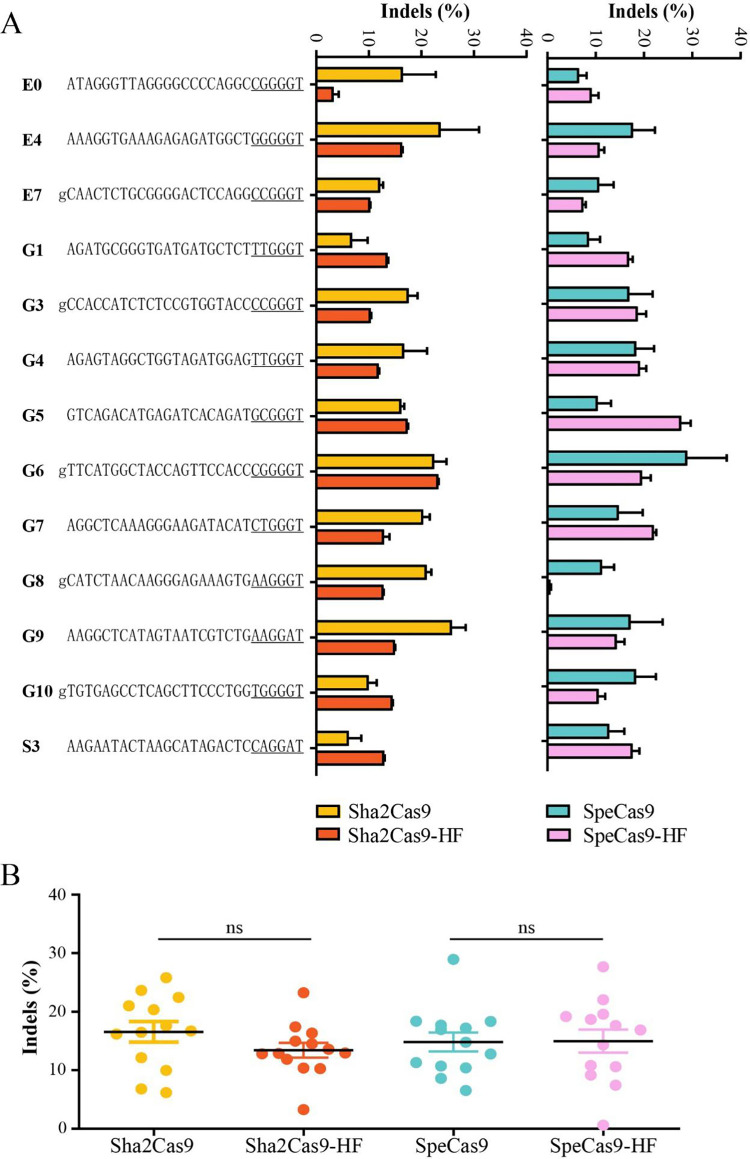
Evaluation of Sha2Cas9-HF and SpeCas9-HF on-target activities. (**A**) Comparison of activities of high-fidelity Cas9s to the wild-type Cas9s (*n =* 3). The target sequences are shown on the left. PAM is underlined. If the first nucleotide is C or T, additional “g” is added for U6 promoter transcription. Underlying data for all summary statistics can be found in S1 Data. (**B**) Quantification of editing efficiency for SaCas9, SmiCas9, Sha2Cas9, and SpeCas9. Underlying data for all summary statistics can be found in S1 Data.

Next, we compared the activities of high-fidelity Cas9s to those of wild-type Cas9s (Sha2Cas9 versus Sha2Cas9-HF; SpeCas9 versus SpeCas9-HF) with a panel of 13 endogenous sites. Western blot analysis revealed that the protein expression levels of high-fidelity Cas9s and wild-type Cas9s were comparable (Fig 3B). All four Cas9s generated indels at targets with varying efficiencies (Fig 5A). Overall, high-fidelity Cas9s and wild-type Cas9s displayed comparable efficiencies (Fig 5B). However, different efficiencies were observed for a number of targets. For example, SpeCas9-HF displayed higher efficiency than SpeCas9 at the G5 site, whereas SpeCas9-HF displayed lower efficiency than SpeCas9 at the G8 site. These data demonstrated that the preference of high-fidelity Cas9s for nucleotides differs from that of wild-type Cas9s for genome editing.

## Discussion

Different nucleotide preferences have been observed among natural Cas9 nucleases. For example, SpCas9 favors G-rich sequences but disfavors T-rich sequences [8]; AsCas12a favors A-rich sequences but disfavors G-rich sequences [9]. One possible strategy to achieve high efficiency of genome editing is to harness multiple natural Cas nucleases for genome editing, and a collection of these nucleases could cover all possible sequences. A number of Cas nucleases, such as SaCas9 [17], NmeCas9 [20], CjCas9 [21], AaCas12b [22], and Cas12f1 [23,24], have been harnessed for genome editing. We previously developed BlatCas9 [25], SauriCas9 [13], SlugCas9 [14], and SchCas9 [15] for genome editing. In this study, we further expanded the Cas repertoire by developing SmiCas9, Sha2Cas9, and SpeCas9. Importantly, they contain a compact genome, facilitating delivery by a single adeno-associated virus (AAV) for in vivo genome editing. These newly developed Cas9s will enhance our ability to achieve high efficiency genome editing.

Different nucleotide preferences have also been observed between natural Cas9s and their engineered variants. We and others previously screened thousands of sgRNA activities for SpCas9 and its engineered variants and observed different nucleotide preferences [8,26]. For example, SpCas9 slightly prefers A and G at sgRNA position 10, while SpCas9-HF1 strongly prefers C at this position [8]. In this study, we generated two high-fidelity versions of Cas9s. Although they only contain 2 or 3 amino acid modifications, distinct nucleotide preferences were observed for a number of targets. Therefore, engineered Cas9s not only change specificity or targeting scope [27–30] but also change nucleotide preferences.

Cas9s with flexible PAMs are crucial for precision positioning. In addition to SpCas9, several other natural Cas nucleases with dinucleotide PAMs have been identified, including FnCas9 [31], Nme2Cas9 [32], SauriCas9 [13], SlugCas9 [14], SchCas9 [15], and AaCas12b [22]. In this study, we identified the serine residue corresponding to SaCas9 N986 associated with the simple NNGG PAM requirement. This PAM occurs, on average, once in every approximately 8 randomly chosen genomic loci. We further identified three amino acids that determined the NNGG PAM requirement of SaCas9. With the continuous expansion of the Cas9 database, our strategy will offer a clue to identify more SaCas9 orthologs with NNGG PAMs.

## Materials and methods

### Cell culture and transfection

HEK293T cells were cultured in DMEM (Gibco) supplemented with 10% FBS (Gibco) and 1× penicillin–streptomycin (Gibco) at 37°C with 5% CO_2_. HEK293T cells were transfected with Lipofectamine 2000 (Life Technologies) according to the manufacturer’s instructions. For Cas9 PAM sequence screening, 1.2 × 10^7^ HEK293T cells were transfected with a total of 10 μg of Cas9 plasmid and 5 μg of sgRNA plasmid in 10-cm dishes. For genome editing comparisons of Cas9, 10^5^ cells were transfected with a total of 300 ng of Cas9 plasmid and 200 ng of sgRNA plasmid in 48-well plates.

### Plasmid construction

Cas9 expression plasmid construction: The plasmid pX601 (Addgene#61591) was amplified by the primers px601-F/px601-R to obtain the pX601 backbone. The human codon–optimized Cas9 gene (S1 Table) was synthesized by HuaGene (Shanghai, China) and cloned into the pX601 backbone by the NEBuilder assembly tool (NEB) according to the manufacturer’s instructions. Sequences of each Cas9 were confirmed by Sanger sequencing (GENEWIZ, Suzhou, China).

sgRNA expression plasmid construction: sgRNA expression plasmids were constructed by ligating sgRNA into the Bsa1-digested hU6-Sa_tracr plasmid. The primer sequences and target sequences are listed in S2 and S3 Tables, respectively.

### PAM sequence analysis

Twenty base-pair sequences (AAGCCTTGTTTGCCACCATG/GTGAGCAAGG GCGAGGAGCT) flanking the target sequence (GAACGGCTCGGAGATCATC ATTGCGNNNNNNN) were used to fix the target sequences. GCG and GTGAGCAAGGGCG AGGAGCT were used to fix a 7-bp random sequence. Target sequences with in-frame mutations were used for PAM analysis. The 7-bp random sequence was extracted and visualized by WebLogo [33] and a PAM wheel chart to identify PAMs [29].

### Genome editing for endogenous sites

HEK293T cells were seeded into 48-well plates and transfected with a total of 300 ng of Cas9 plasmid and 200 ng of sgRNA plasmid by Lipofectamine 2000 (1 μL). Cells were collected 5 days after transfection. Genomic DNA was isolated, and the target sites were PCR amplified and extracted by QuickExtract DNA Extraction Solution (Epicentre) for deep sequencing. For genomic HEK293T DNA, the PCR products were subjected to a T7E1 assay to check the editing efficiency. The primer sequences are listed in S2 Table.

### Test of Cas9 specificity

To test the specificity of Cas9, we generated two GFP reporter cell lines with the CTGG PAM. The cells were seeded into 48-well plates and transfected with 300 ng of Cas9 plasmids and 200 ng of sgRNA plasmids by using Lipofectamine 2000. Five days after editing, the GFP-positive cells were analyzed on a Calibur instrument (BD). The data were analyzed using FlowJo.

### GUIDE-seq

GUIDE-seq experiments were performed as described previously [19], with minor modifications. Briefly, 2 × 10^5^ HEK293T cells were transfected with 500 ng of SchCas9/Sa-SchCas9, 500 ng of sgRNA plasmids, and 100 pmol of annealed GUIDE-seq oligonucleotides by electroporation and then seeded into 6 wells. The electroporation voltage, width, and the number of pulses were 1,150 V, 30 ms, and 1 pulse, respectively. Genomic DNA was extracted with the DNeasy Blood and Tissue kit (QIAGEN) 6 days after transfection according to the manufacturer’s protocol. The genome library was prepared and subjected to deep sequencing [19].

### Western blotting

One day before transfection, HEK293T cells were seeded into a 6-well plate. For each well, 2 μg of Cas9-expressing plasmid were transfected using 4 μL of Lipofectamine2000. Three days after transfection, cell samples were collected and total proteins were extracted using NP-40 buffer (Beyotime) supplemented with 1 mM phenylmethanesulfonyl fluoride (PMSF) (Beyotime). The protein was separated by SDS-PAGE gel and transferred onto polyvinylidene fluoride (PVDF) (Thermo) membrane. After transfer, the membrane was blocked with 5% (wt./vol.) BSA (Sigma) in TBS-T (0.1% Tween 20 in 1× TBS) buffer and then incubated in the primary antibody (anti-HA tag (1:1,000; ab236632, Abcam) and anti-GAPDH (1:2,000; 5174s, Cell Signaling) at 4°C overnight. Wash membrane three times in TBS-T for 5 min each time. The second antibody (1:10,000; ab6721, Abcam) was incubated for 1 h at room temperature, and then washed three times and imaged.

### Statistical analysis

All the data are shown as mean ± SD. Statistical analyses were performed using Microsoft Excel. Two-tailed, paired Student *t* tests were used to determine statistical significance when comparing two groups, whereas analyses of variance (ANOVAs) are used for comparisons between for three or more groups. A value of *P* < 0.05 was considered to be statistically significant (*P < 0.05, **P < 0.01, ***P < 0.001).

## Supporting information

S1 Fig Genetic locus of CRISPR/Cas9. (**A**) The structures of CRISPR loci for six SaCas9 orthologs. (**B**) Alignment of CRISPR repeat sequences for six SaCas9 orthologs. (**C**) Alignment of tracrRNA for six SaCas9 orthologs.
(TIF)

S2 Fig Analysis of sgRNAs. (**A**) Alignment of sgRNA scaffolds for six SaCas9 orthologs. The GAAA linker are indicated by the black box. (**B**) Analysis of SaCas9 orthologs’ secondary RNA structures. These structures were generated by an online tool named RNAfold WebServer (http://rna.tbi.univie.ac.at/cgi-bin/RNAWebSuite/RNAfold.cgi).
(TIF)

S3 Fig Analysis of the SaCas9 variant PAMs. (**A**) Amino acid sequence of the SaCas9 variant PI domains. The residues that are important for PAM recognition are marked at the top; the mutations are highlighted in red. (**B**) SaCas9 variant PAMs were analyzed by the GFP activation assay. WebLogos generated by analyzing the deep sequencing data.
(TIF)

S4 Fig Evaluation of the genome editing efficiency of 6 SaCas9 orthologs. (**A**) Examples of the gel pictures of T7EI assay for Sha2Cas9 and SpeCas9. Cleaved fragments are marked by red triangles. Indel frequencies are shown below. Underlying data for all summary statistics can be found in S1 Data. (**B**) Quantification of editing efficiency for 6 SaCas9 orthologs. Underlying data for all summary statistics can be found in S1 Data.
(TIF)

S5 Fig Analysis of four Cas9 ortholog specificity. Schematic of the GFP activation assay for specificity analysis is shown on the top. A panel of sgRNAs with dinucleotide mutations is shown below. sgRNA activities were measured based on GFP expression. Cells without Cas9 transfection were used as a negative control (NC). Mismatches are shown in red (*n =* 3). Underlying data for all summary statistics can be found in S1 Data.
(TIF)

S6 Fig Protein sequence alignment of SaCas9, Sha2Cas9, and SpeCas9. The amino acid residues important for specificity are indicated by vertical lines above. The amino acid residue positions are shown on the right.
(TIF)

S7 Fig Specificity of four Sha2Cas9 variants. (**A**) Schematic of Sha2Cas9 structure. The amino acid residues important for specificity are shown below. (**B**) Test of four Sha2Cas9 variant specificity. Schematic of the GFP activation assay for specificity analysis is shown on the top. A panel of sgRNAs with dinucleotide mutations is shown below. sgRNA activities were measured based on GFP expression. Cells without Cas9 transfection were used as a negative control (NC). Mismatches are shown in red (*n =* 2 or 3). Underlying data for all summary statistics can be found in S1 Data.
(TIF)

S8 Fig Specificity of four SpeCas9 variants. (**A**) Schematic of SpeCas9 structure. The amino acid residues important for specificity are shown below. (**B**) Test of four SpeCas9 variant specificity. Schematic of the GFP activation assay for specificity analysis is shown on the top. A panel of sgRNAs with dinucleotide mutations is shown below. sgRNA activities were measured based on GFP expression. Cells without Cas9 transfection were used as a negative control (NC). Mismatches are shown in red (*n* = 3). Underlying data for all summary statistics can be found in S1 Data.
(TIF)

S1 Data Underlying values for all reported summary statistics. Raw data from all reported summary statistics.
(XLSX)

S1 Table The Cas9 ID and human codon–optimized Cas9 gene. The file contains the Cas9 ID, host strain, tracrRNA, and amino acid sequences of SaCas9 orthologs used in this study. The human codon–optimized Cas9 genes were synthesized.
(DOCX)

S2 Table Primers used in this study. A list of oligonucleotide pairs and primers used for deep sequencing.
(DOCX)

S3 Table Target sites used in this study. A list of the endogenous target sites of human and their downstream PAM. PAM, protospacer adjacent motif.
(DOCX)

S1 Raw Images Raw images of Figs 3 and S4. (JPG)

## Abbreviations

AAV: adeno-associated virus
ANOVA: analysis of variance
CRISPR: Clustered Regularly Interspaced Short Palindromic Repeats
crRNA: CRISPR RNA
indels: insertions or deletions
PAM: protospacer adjacent motif
PI: PAM-interacting
PMSF: phenylmethanesulfonyl fluoride
PVDF: polyvinylidene fluoride
sgRNA: single-guide RNA
tracrRNA: *trans*-activating crRNA
