# Correction: Identification of SaCas9 orthologs containing a conserved serine residue that determines simple NNGG PAM recognition

**DOI:** 10.1371/journal.pbio.3003036

**Published:** 2025-03-13

**Authors:** Shuai Wang, Chen Tao, Huilin Mao, Linghui Hou, Yao Wang, Tao Qi, Yuan Yang, Sang-Ging Ong, Shijun Hu, Renjie Chai, Yongming Wang

There is an error in Fig 1. The image labeled ’No Cas9’ in Fig 1C is incorrect. Please see the correct Fig 1 here.

**Fig 1:**
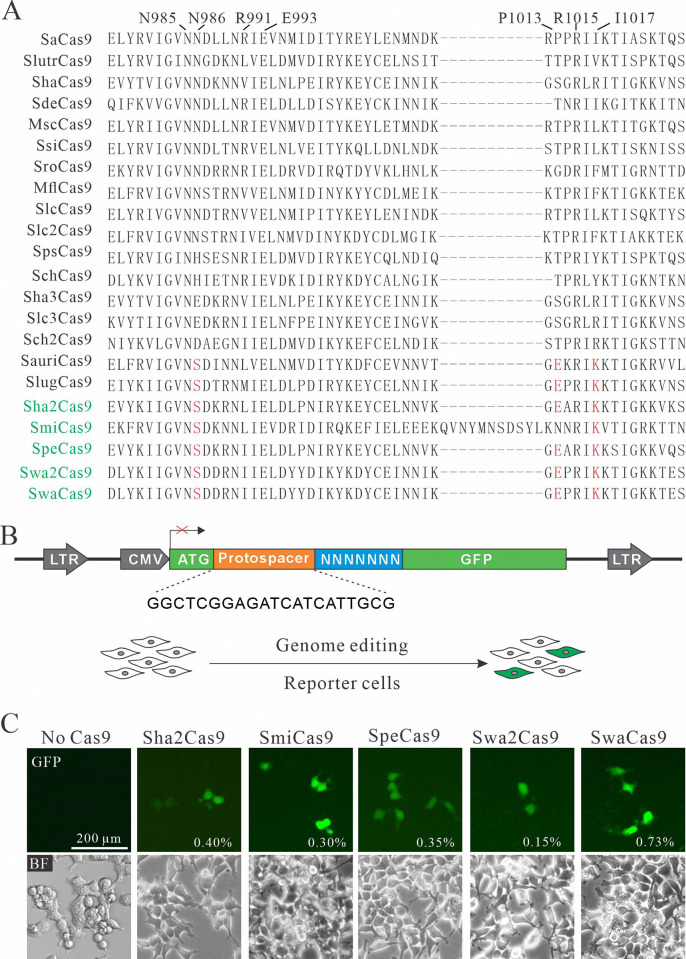
Analysis of five SaCas9 ortholog activities. (**A**) Amino acid sequences of the SaCas9 ortholog PI domain are aligned. The residues that are important for PAM recognition are indicated at the top; the conserved residues among newly identified SaCas9 orthologs are shown in red; the names of newly identified Cas9s are shown in green. (**B**) Design of the GFP activation reporter construct. A target sequence (protospacer) containing a 7-bp random sequence is inserted between ATG and the GFP-coding sequence. The library DNA is stably integrated into HEK293T cells by lentivirus. (**C**) Transfection of SaCas9 orthologs induced GFP expression. Percentage of GFP-positive cells was shown. The cells without transfection of Cas9 were used as a negative control.
