# Supplementary figures and images for: Identification of SaCas9 orthologs containing a conserved serine residue that determines simple NNGG PAM recognition
Source: PLoS Biol. 2022 Nov 30;20(11):e3001897. doi: 10.1371/journal.pbio.3001897 (PMC9710800; doi:10.1371/journal.pbio.3001897)

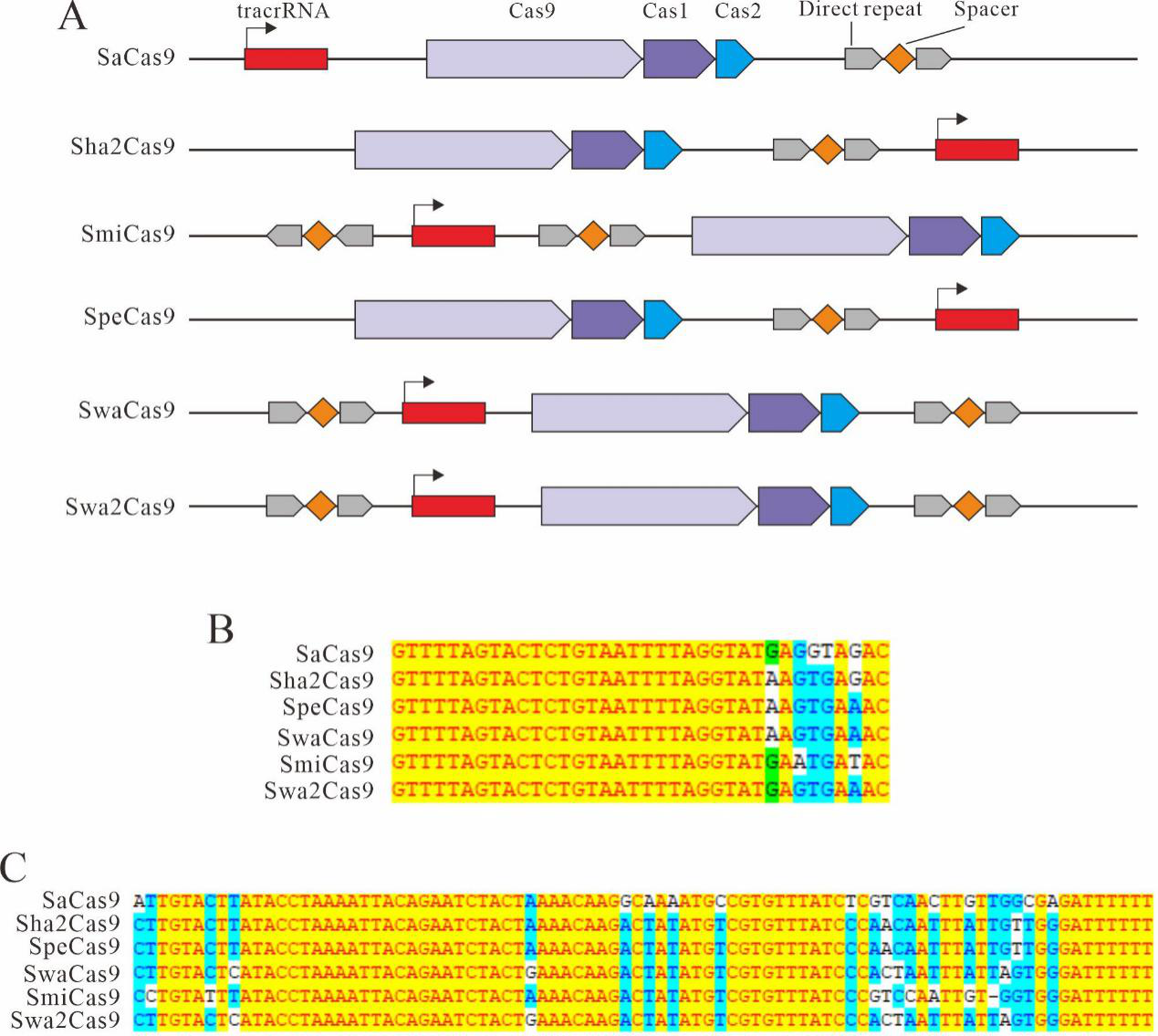

Supplement: S1 Fig — (A) The structures of CRISPR loci for six SaCas9 orthologs. (B) Alignment of CRISPR repeat sequences for six SaCas9 orthologs. (C) Alignment of tracrRNA for six SaCas9 orthologs. (TIF) [file pbio.3001897.s001.tif]

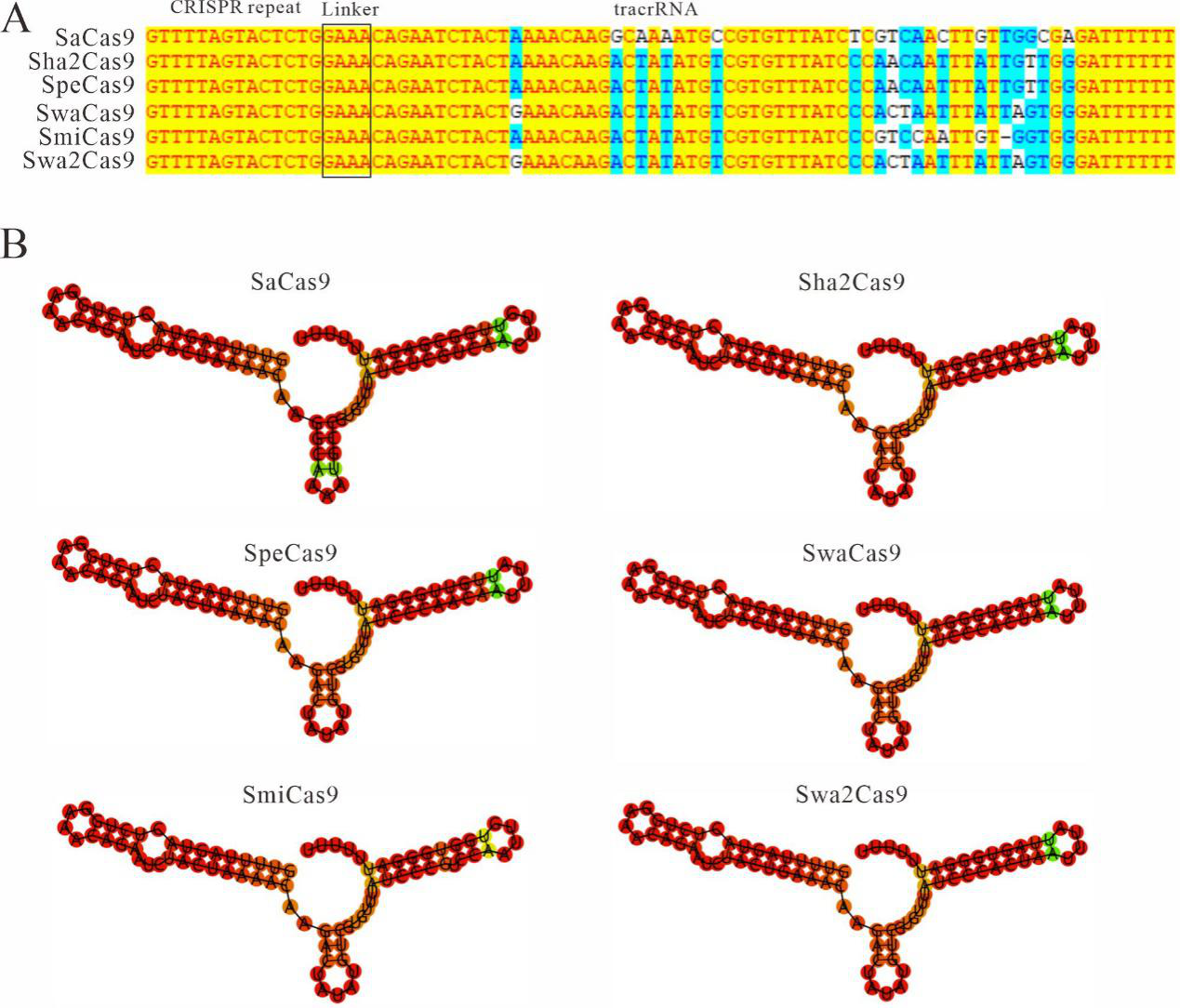

Supplement: S2 Fig — (A) Alignment of sgRNA scaffolds for six SaCas9 orthologs. The GAAA linker are indicated by the black box. (B) Analysis of SaCas9 orthologs’ secondary RNA structures. These structures were generated by an online tool named RNAfold WebServer (http://rna.tbi.univie.ac.at/cgi-bin/RNAWebSuite/RNAfold.cgi). (TIF) [file pbio.3001897.s002.tif]

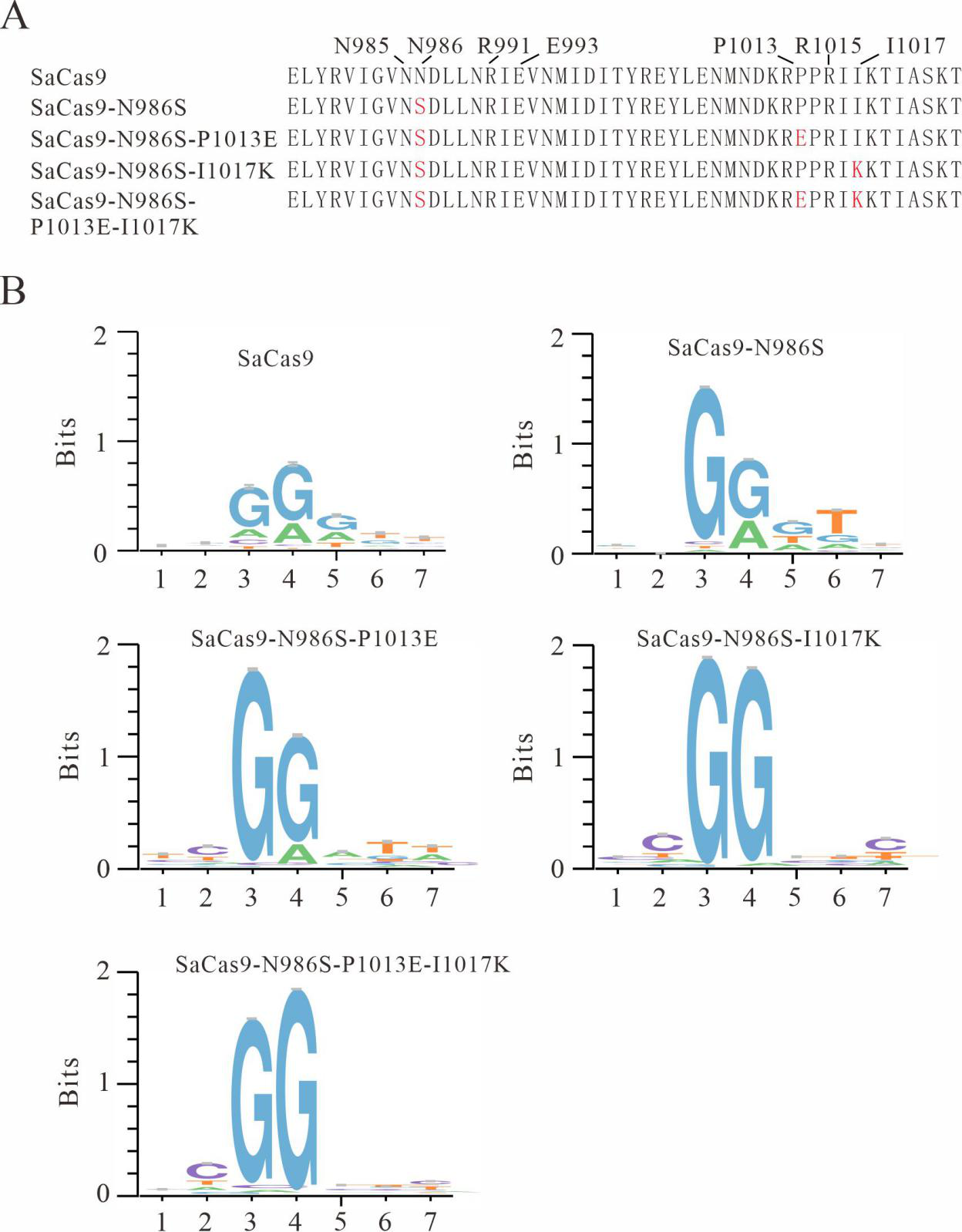

Supplement: S3 Fig — (A) Amino acid sequence of the SaCas9 variant PI domains. The residues that are important for PAM recognition are marked at the top; the mutations are highlighted in red. (B) SaCas9 variant PAMs were analyzed by the GFP activation assay. WebLogos generated by analyzing the deep sequencing data. (TIF) [file pbio.3001897.s003.tif]

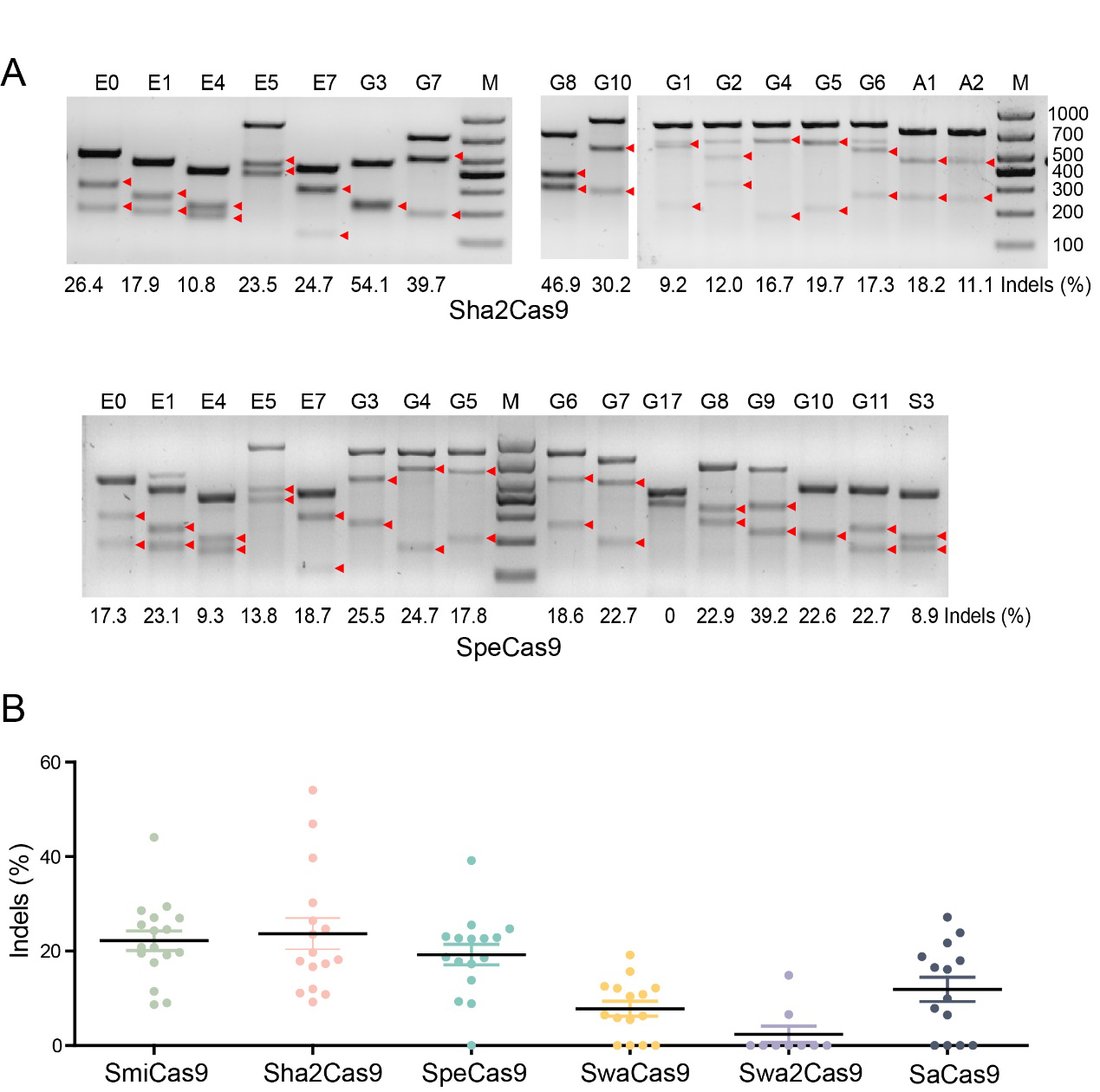

Supplement: S4 Fig — (A) Examples of the gel pictures of T7EI assay for Sha2Cas9 and SpeCas9. Cleaved fragments are marked by red triangles. Indel frequencies are shown below. Underlying data for all summary statistics can be found in S1 Data. (B) Quantification of editing efficiency for 6 SaCas9 orthologs. Underlying data for all summary statistics can be found in S1 Data. (TIF) [file pbio.3001897.s004.tif]

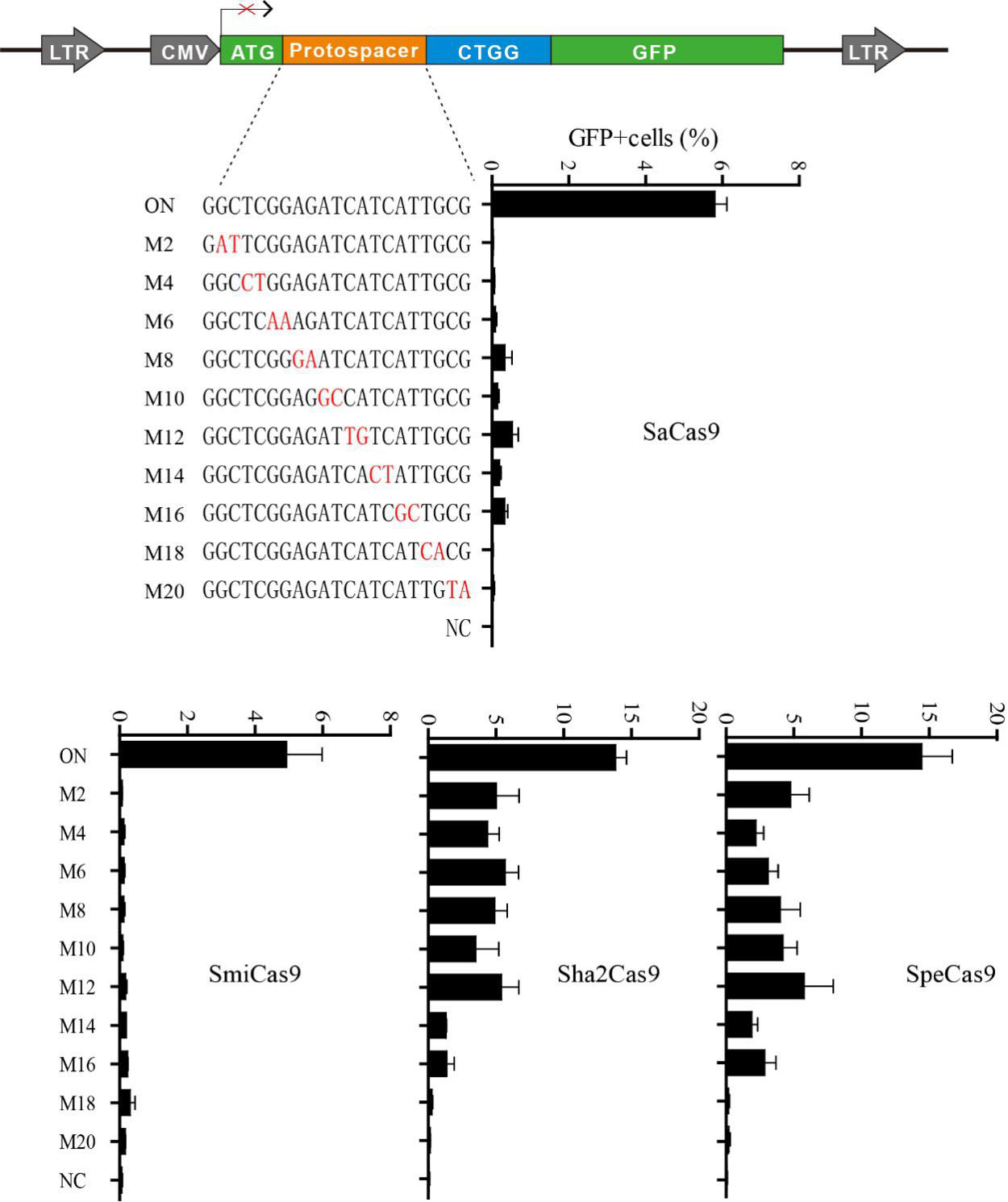

Supplement: S5 Fig — Schematic of the GFP activation assay for specificity analysis is shown on the top. A panel of sgRNAs with dinucleotide mutations is shown below. sgRNA activities were measured based on GFP expression. Cells without Cas9 transfection were used as a negative control (NC). Mismatches are shown in red (n = 3). Underlying data for all summary statistics can be found in S1 Data. (TIF) [file pbio.3001897.s005.tif]

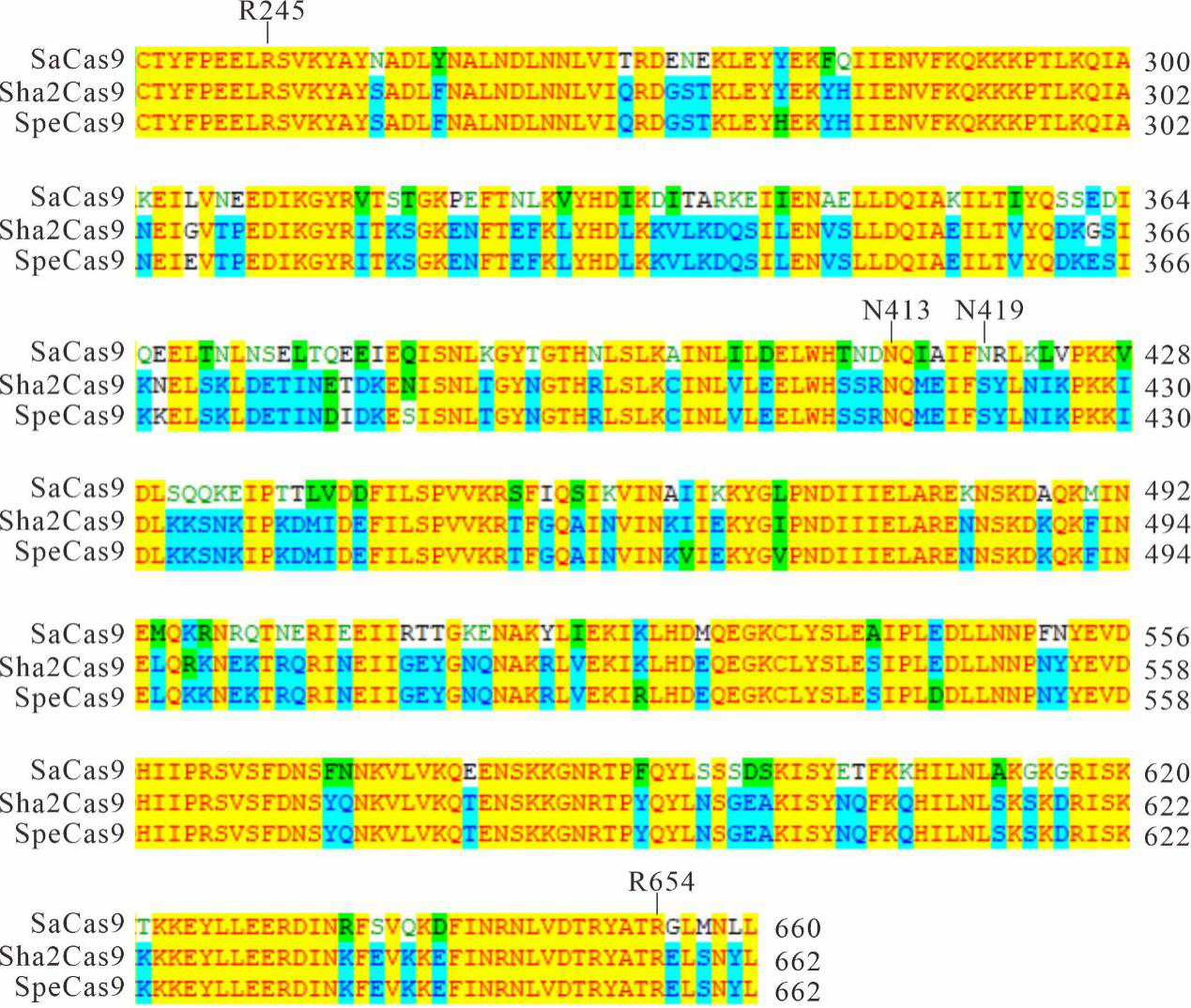

Supplement: S6 Fig — The amino acid residues important for specificity are indicated by vertical lines above. The amino acid residue positions are shown on the right. (TIF) [file pbio.3001897.s006.tif]

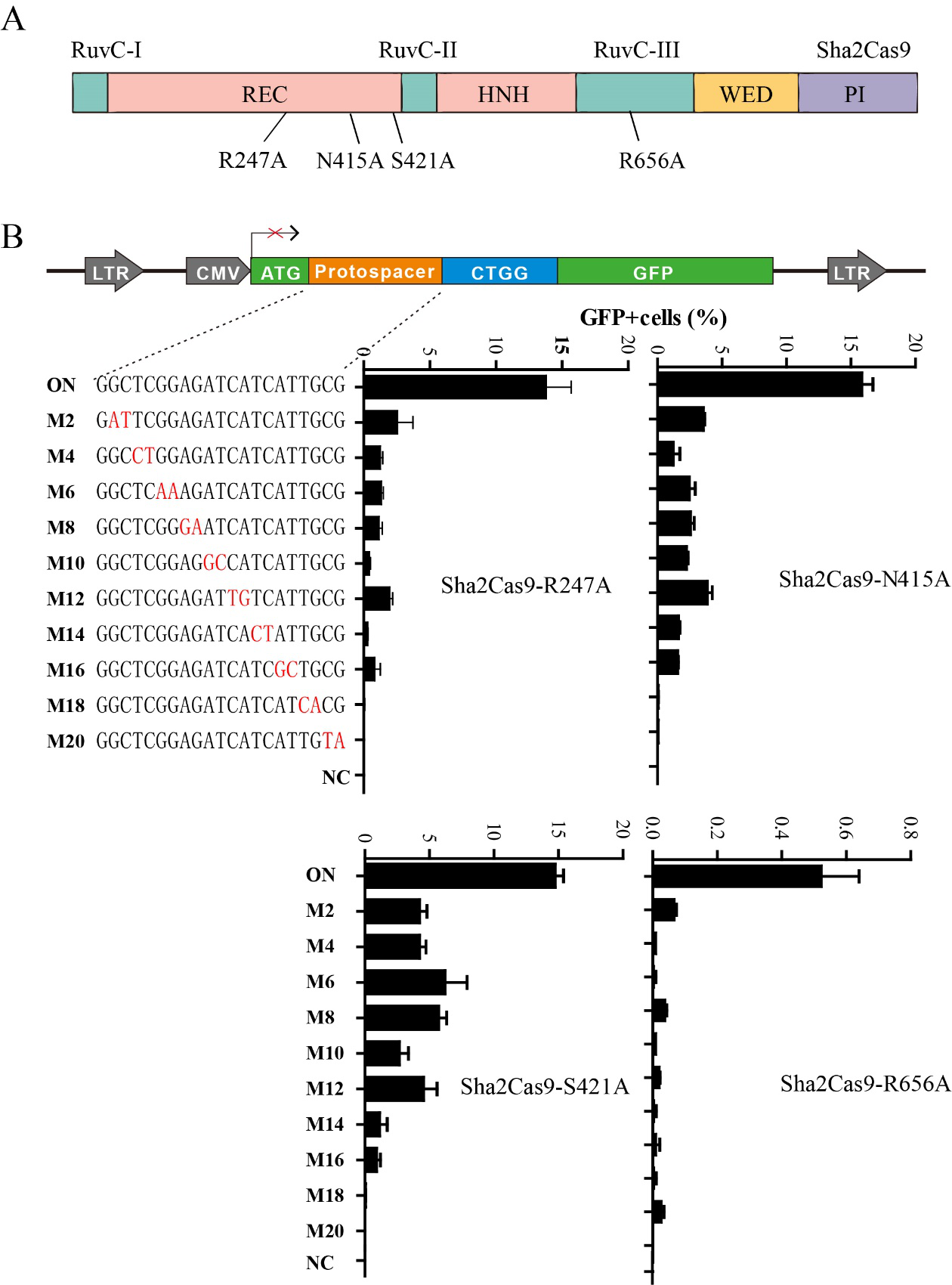

Supplement: S7 Fig — (A) Schematic of Sha2Cas9 structure. The amino acid residues important for specificity are shown below. (B) Test of four Sha2Cas9 variant specificity. Schematic of the GFP activation assay for specificity analysis is shown on the top. A panel of sgRNAs with dinucleotide mutations is shown below. sgRNA activities were measured based on GFP expression. Cells without Cas9 transfection were used as a negative control (NC). Mismatches are shown in red (n = 2 or 3). Underlying data for all summary statistics can be found in S1 Data. (TIF) [file pbio.3001897.s007.tif]

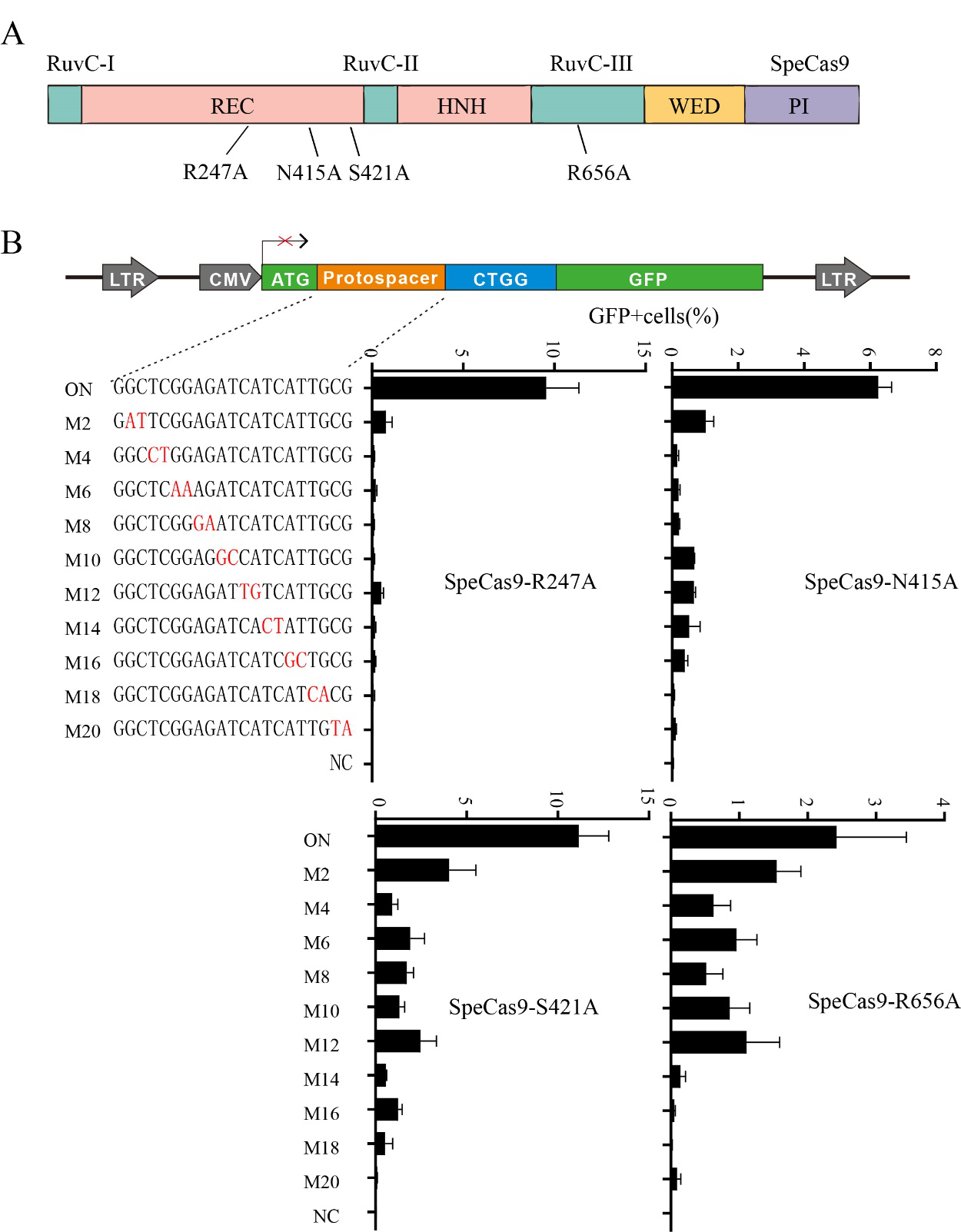

Supplement: S8 Fig — (A) Schematic of SpeCas9 structure. The amino acid residues important for specificity are shown below. (B) Test of four SpeCas9 variant specificity. Schematic of the GFP activation assay for specificity analysis is shown on the top. A panel of sgRNAs with dinucleotide mutations is shown below. sgRNA activities were measured based on GFP expression. Cells without Cas9 transfection were used as a negative control (NC). Mismatches are shown in red (n = 3). Underlying data for all summary statistics can be found in S1 Data. (TIF) [file pbio.3001897.s008.tif]

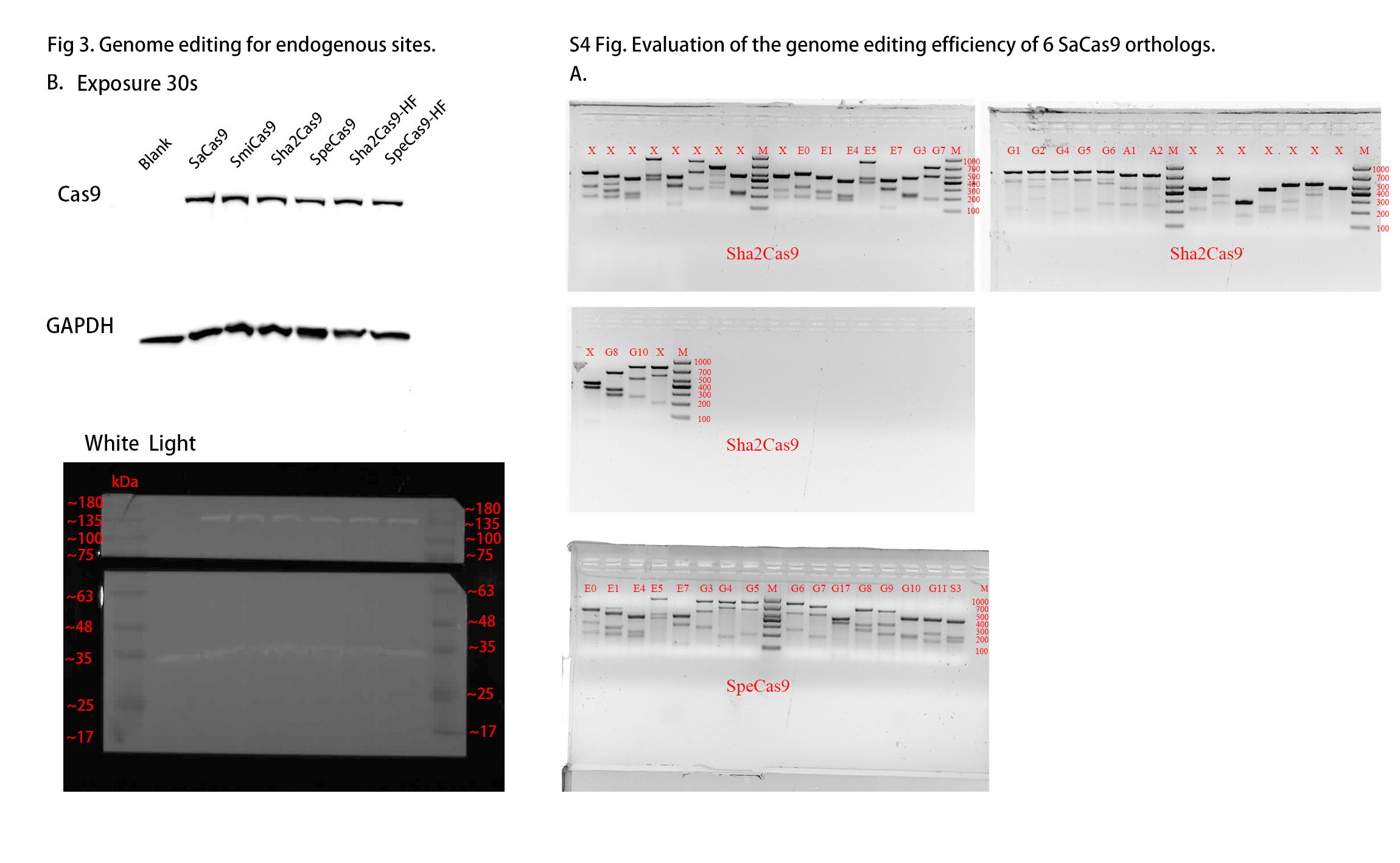

Supplement: S1 Raw Images — (JPG) [file pbio.3001897.s013.jpg]
