# Supplementary material for: Identification of SaCas9 orthologs containing a conserved serine residue that determines simple NNGG PAM recognition
Source: PLoS Biol. 2022 Nov 30;20(11):e3001897. doi: 10.1371/journal.pbio.3001897 (PMC9710800; doi:10.1371/journal.pbio.3001897)
Supplement: S1 Table — The file contains the Cas9 ID, host strain, tracrRNA, and amino acid sequences of SaCas9 orthologs used in this study. The human codon–optimized Cas9 genes were synthesized. (DOCX) [file pbio.3001897.s010.docx]

| **Table S1** | |
| --- | --- |
| Cas9 ID in NCBI | WP_107532850 |
| Host strain | Staphylococcus warneri |
| Nuclease name | SwaCas9 |
| Human codon-optimized Cas9_Gene | ATGAAAGAGAAGTATATCTTAGGACTGGATCTGGGAATCACCTCTGTGGGCTACGGAATTATCAACTTCGAGACAAAGAAAATCATTGACGCCGGCGTGCGGCTGTTCCCCGAGGCCAATGTGGACAACAATGAGGGCAGACGGAGCAAGCGGGGTTCTAGAAGACTGAAGCGCCGGCGGATTCACAGACTAGATAGAGTGAAGAGCCTGCTCACCGAGTACAATCTGATCAACAGAGAACAGATCCCCACAAGCAACAATCCCTACCAGATAAGAGTGAAAGGACTGTCCGAGATTCTGAGCAAAGATGAACTGGCTATCGCCCTGCTGCATCTGGCCAAGAGACGCGGCATCCACAACATCAATGTGTCCAGCGAGGATGAGGACGCCAGCAACGAGCTGAGCACCAAGGAGCAGATCAATCGGAACAACAAGCTGCTGAAGAACAAATACGTGTGTGAAGTGCAGCTGCAAAGACTGAAAGAAGGCCAGATCCGGGGCGAAAAGAACAGATTCAAGACAACCGACATCCTGAAGGAAATCGATCAGCTGCTTAAGGTGCAGAAGGATTACCACAACCTGGATATCGACTTCATCAACCAGTACAAGGAGATCGTGGAAACCAGAAGGGAATACTTTGAAGGGCCTGGACAAGGCAGCCCCTTCGGCTGGAACGGAGATCTGAAGAAGTGGTACGAGATGCTGATGGGCCACTGCACCTACTTCCCTCAGGAGCTGCGGAGCGTGAAGTATGCCTACTCGGCCGACCTGTTTAACGCCCTGAACGATTTAAACAACCTTATCATCCAGAGAAATGACTCTGAAAAGCTGGAGTACCACGAGAAGTACCACATCATCGAGAACGTGTTTAAGCAGAAGAAGAAGCCTACCCTGAAGCAAATCGCTAAAGAAATCGGCGTGAACCCTGAGGACATCAAAGGCTACCGGATCACGAAGAGCGGCACCCCACAGTTCACCGAATTCAAGCTGTACCACGACCTGAAGTCTATCGTGTTTGACAAGAGCATCCTGGAGAACGAGGCCATCCTGGACCAGATCGCCGAAATCCTGACAATTTACCAAGACGAGGAATCTATCAAGGAAGAGCTGAACAAACTGCCTGAGATCCTGAACGAACAGGACAAAGCTGAAATCGCCAAGCTGACAGGCTATAATGGCACACACAGACTGAGCCTGAAGTGCATCCATCTGATCAATGAAGAGCTGTGGCAGACCAGCAGAAATCAGATGGAAATTTTCAACTATCTGAACATCAAGCCTAACAAGGTGGATCTCAGCGAGCAGAACAAAATCCCCAAGGACCTGGTTGATGAGTTCATCCTGAGTCCTGTGGTGAAACGGACCTTTATCCAGAGCATCAACGTGATCAACAAGGTGATCGAGAAGTACGGCATCCCTGAGGATATCATCATCGAGCTGGCTAGAGAGAACAACAGCGACGACAGAAAGAAATTCATCAACAACCTGCAGAAAAAGAACGAGGCTACCAGAAAGAGAATAAATGAGATCATCGGACAGACCGGCAACCAAAACGCTAAACGGATCGTGGAAAAGATTCGGCTGCACGATCAGCAGGAGGGAAAATGCCTGTACTCTCTGGAAAGCATCCCTCTGATGGACCTGCTGAACAATCCACAGAACTACGAAGTGGACCACATTATCCCGAGAAGCGTGGCCTTCGATAACTCTATCCACAACAAGGTGCTGGTGAAGCAGATCGAGAACAGCAAGAAAGGCAACAGAACCCCTTACCAGTACCTGAACAGCAGCGACGCAAAGCTGTCTTACAATCAGTTTAAGCAGCACATCCTGAACCTGAGCAAATCTAAGGACAGAATCAGCAAGAAGAAGAAGGACTACCTGCTGGAAGAGAGAGACATCAACAAGTTCGAGGTGCAGAAGGAATTCATCAACAGAAACCTGGTGGACACCCGGTACGCCACCAGAGAGCTAACGAGCTACCTGAAAGCCTACTTCTCCGCCAACAACATGGACGTCAAGGTAAAGACAATCAACGGCAGCTTCACCAACCACCTGAGAAAGGTGTGGCGATTTGACAAGTACCGGAACCACGGCTACAAGCACCACGCCGAGGATGCCCTGATCATCGCCAACGCCGATTTCCTGTTCAAAGAGAACAAGAAGCTGCAGAACGCCAACAAAATCCTCGAGAAACCTACAATCGAGAACAACACCAAGAAGGTGACCGTGGAAAAAGAGGAAGACTATAACAATATGTTCGAGACACCTAAACTGGTGGAAGATATCAAGCAGTACAGAGACTATAAGTTCAGCCACCGCGTGGACAAGAAGCCCAACAGACAGCTGATCAATGATACCCTGTATTCAACAAGAATGAAGGACGAGCACGACTACATCGTGCAGACCATCACAGACATCTACGGCAAGGACAATACCAACCTTAAGAAACAGTTCAACAAGAACCCTGAGAAGTTCCTGATGTACCAGAACGACCCTAAAACCTTCGAGAAGCTGTCCATCATCATTAAGCAGTACAGCGATGAGAAAAACCCACTGGCTAAGTACTACGAGGAAACCGGCGAGTACCTGACTAAGTACAGCAAGAAGAACAATGGCCCCATCGTCAAGAAGATCAAGTTACTGGGCAATAAGGTCGGCAACCATCTGGACGTTACCAAAAAGTACGAGAATTCTACAAAAAAGCTGGTTAAGCTGAGCATCAAGAATTACCGGTTTGACGTGTACCTGACAGAGAAGGGCTACAAGTTCGTGACCATCGCCTACCTGAACGTGTTCAAGAAGGACAACTACTACTACATCCCAAAGGACATGTACCAGGAGCTGAAGGCCAAGAAGAAGATAAAGGACACCGACCAATTCATCGCCTCTTTCTACAAGAATGACCTGATTAAGCTGAACGGCGACCTGTACAAAATAATCGGCGTCAACTCCGATGACCGGAACATCATCGAACTGGACTATTACGACATCAAGTACAAGGATTACTGCGAGATCAACAACATCAAGGGCGAGCCTAGAATCAAGAAAACAATTGGGAAGAAGACCGAGTCCATCGAAAAACTGACGACCGACGTGTTGGGCAACCTGTACCTGCACAGCACAGAGAAGGCCCCTCAGCTGATCTTCAAAAGAGGCCTG |
| CAS9 Amino Acid | MKEKYILGLDLGITSVGYGIINFETKKIIDAGVRLFPEANVDNNEGRRSKRGSRRLKRRRIHRLDRVKSLLTEYNLINREQIPTSNNPYQIRVKGLSEILSKDELAIALLHLAKRRGIHNINVSSEDEDASNELSTKEQINRNNKLLKNKYVCEVQLQRLKEGQIRGEKNRFKTTDILKEIDQLLKVQKDYHNLDIDFINQYKEIVETRREYFEGPGQGSPFGWNGDLKKWYEMLMGHCTYFPQELRSVKYAYSADLFNALNDLNNLIIQRNDSEKLEYHEKYHIIENVFKQKKKPTLKQIAKEIGVNPEDIKGYRITKSGTPQFTEFKLYHDLKSIVFDKSILENEAILDQIAEILTIYQDEESIKEELNKLPEILNEQDKAEIAKLTGYNGTHRLSLKCIHLINEELWQTSRNQMEIFNYLNIKPNKVDLSEQNKIPKDLVDEFILSPVVKRTFIQSINVINKVIEKYGIPEDIIIELARENNSDDRKKFINNLQKKNEATRKRINEIIGQTGNQNAKRIVEKIRLHDQQEGKCLYSLESIPLMDLLNNPQNYEVDHIIPRSVAFDNSIHNKVLVKQIENSKKGNRTPYQYLNSSDAKLSYNQFKQHILNLSKSKDRISKKKKDYLLEERDINKFEVQKEFINRNLVDTRYATRELTSYLKAYFSANNMDVKVKTINGSFTNHLRKVWRFDKYRNHGYKHHAEDALIIANADFLFKENKKLQNANKILEKPTIENNTKKVTVEKEEDYNNMFETPKLVEDIKQYRDYKFSHRVDKKPNRQLINDTLYSTRMKDEHDYIVQTITDIYGKDNTNLKKQFNKNPEKFLMYQNDPKTFEKLSIIIKQYSDEKNPLAKYYEETGEYLTKYSKKNNGPIVKKIKLLGNKVGNHLDVTKKYENSTKKLVKLSIKNYRFDVYLTEKGYKFVTIAYLNVFKKDNYYYIPKDMYQELKAKKKIKDTDQFIASFYKNDLIKLNGDLYKIIGVNSDDRNIIELDYYDIKYKDYCEINNIKGEPRIKKTIGKKTESIEKLTTDVLGNLYLHSTEKAPQLIFKRGL |
| Lengh(aa) | 1054 |
| repeat | GTTTTAGTACTCTGTAATTTTAGGTATAAGTGAAAC |
| tracrRNA | TTGTACTCATACCTAAAATTACAGAATCTACTGAAACAAGACTATATGTCGTGTTTATCCCACTAATTTATTAGTGGGATTTTTT |
| sgRNA | GTTTTAGTACTCTGgaaaCAGAATCTACTGAAACAAGACTATATGTCGTGTTTATCCCACTAATTTATTAGTGGGATTTTTT |

| Cas9 ID in NCBI | WP_044361501 |
| --- | --- |
| Host strain | Staphylococcus microti |
| Nuclease name | SmiCas9 |
| Human codon-optimized Cas9_Gene | ATGGAAAAGGATTACATCCTGGGCCTGGATATCGGCATCGGATCCGTGGGATACGGCCTGATTGATTACGATACCAAAAGCATCATTGACGCCGGCGTGCGGCTGTTTCCAGAGGCTAACGCTGACAACAACCTGGGCAGACGGGCCAAAAGAGGTGCTAGACGGCTGAAGCGGCGGAGAATCCACAGGCTGGAGCGCGTCAAGTCCCTGCTGTCTGAATACAAGATCATCTCTGGCCTGGCCCCAACAAACAACCAGCCTTACAACATCAGAGTGAAAGGACTGACCGAGCAGCTGACAAAAGATGAGCTCGCTGTGGCCCTGCTCCATATCGCCAAACGGAGAGGAATCCACAACGTGGACGTTGCTGCCGACAAGGAAGAGACAGCCAGCGACAGCCTGAGCACCAAGGACCAAATCAACAAAAATGCGAAGTTTCTAGAATCCAGATACGTGTGCGAGCTCCAGAAAGAGAGACTGGAAAACGAAGGCCACGTGCGGGGCGTGGAAAACAGATTCCTGACAAAGGACATCGTTAGAGAAGCTAAGAAGATTATCGATACACAGATGCAGTACTACCCCGAAATCGACGAAACCTTCAAGGAGAAATACATCTCTCTGGTGGAAACAAGAAGAGAGTACTACGAGGGCCCTGGCAAGGGCTCTCCTTACGGCTGGGACGCCGATGTGAAAAAGTGGTACCAGCTGATGATGGGCCATTGTACCTACTTCCCCGTGGAGTTCAGATCTGTGAAGTACGCCTATACGGCCGATCTGTACAATGCCCTGAACGACCTGAACAACCTGACCATCGCTCGTGATGACAACCCCAAGCTGGAATACCACGAGAAGTACCATATCATTGAAAACGTGTTCAAGCAGAAGAGGAATCCCACACTGAAGCAGATTGCCAAAGAGATCGGCGTGAACGACATCAACATCTCTGGCTACAGAGTGACCAAGTCTGGAAAACCTCAATTTACCTCTTTCAAACTGTTCCACGATCTCAAGAAGGTCGTGAAGGACCACGCCATCCTGGACGACATCGACCTGTTGAATCAGATCGCCGAGATCCTGACGATCTACCAGGACAAGGATAGCATCGTGGCCGAGCTGGGCCAGCTGGAATACCTGATGAGCGAAGCCGATAAGCAGAGCATCTCTGAACTTACCGGCTATACCGGCACCCACAGCCTGAGCCTGAAGTGCATGAATATGATCATTGATGAGCTGTGGCACAGCAGCATGAACCAGATGGAAGTGTTCACATACCTGAACATGCGGCCTAAGAAGTATGAGCTGAAAGGCTACCAAAGAATCCCCACCGACATGATCGATGACGCCATTCTGAGCCCTGTGGTGAAGCGCAGCTTTAAACAGGCCATCGGCGTGGTCAACGCCATCATCAAGAAGTACGGCCTGCCTAAGGACATCATCATCGAGCTGGCCAGAGAGTCTAACAGCGCCGAGAAGAGCCGGTACCTGAGAGCCATCCAAAAAAAGAACGAGAAGACCAGAGAGCGGATTGAGGCCATTATCAAGGAGTACGGCAACGAGAATGCCAAGGGCCTGGTGCAGAAGATCAAACTGCACGACGCCCAGGAGGGAAAGTGCCTGTACAGCCTGAAGGACATACCTCTGGAGGATCTGCTGAGAAACCCTAACAATTACGACATCGACCACATCATCCCTAGAAGCGTCTCCTTCGATGACAGCATGCACAACAAGGTGCTGGTGAGGAGGGAACAGAACGCTAAGAAGAACAACCAGACCCCATACCAGTACCTGACCAGCGGATACGCCGATATCAAGTACAGCGTGTTCAAGCAGCACGTGCTGAACCTGGCTGAAAACAAGGATAGAATGACTAAGAAAAAGAGAGAGTACCTGCTGGAGGAGCGGAACATCAACAAGTACGACGTGCAGAAAGAATTCATCAATCGGAACCTGGTGGACACAAGATACACCACCCGCGAACTGACAACCCTGCTGAAGACCTACTTCACAATCAACAACCTGGACGTGAAGGTGAAGACCATCAACGGCAGCTTTACAGACTTCCTGAGAAAGAGATGGGGCTTCAAAAAGAACCGGGACGAGGGGTACAAACACCACGCGGAAGATGCCCTGATCATCGCAAATGCCGACTATCTGTTCAAGGAACACAAGCTGCTGAAGGAAATCAAGGACGTGTCCGACCTGGCTGGCGACGAGAGAAACAGTAACGTGAAGGATGAAGATCAGTACGAGGAGGTGTTCGGCGGCTATTTCAAGATCGAAGACATCAAGAAATATAAGATCAAGAAGTTCAGCCACAGAGTGGACAAGAAGCCCAACCGGCAGCTGATCAACGATACAATCTATTCTACCCGGGTGAAGGACGATAAAAGATACCTTATTAACACCCTGAAAAATCTGTACGACAAGAGCAACGGCGACCTGAAGGAGCGGATGCAGAAAGACCCTGAGAGCCTTCTGATGTACCACCACGATCCTCAGACGTTCGAGAAGTTAAAAATCGTGATGAGCCAGTACGAGAATGAGAAGAACCCCCTGGCCAAGTATTTTGAGGAGACAGGACAGTACCTGACCAAGTACGCCAAGCACGATAACGGCCCTGCCATCCACAAGATCAAATATTACGGCAACAAGCTGGTGGAGCACCTGGACATCACAAAGAATTACCACAATCCTCAGAACAAGGTCGTGCAGCTGTCCCAGAAGAGCTTCAGATTCGACGTGTACCAGACCGACAAGGGCTACAAATTCATCAGCATCGCCTACCTGACCCTGAAGAACGAAAAGAACTACTACGCCATCAGTCAGGAGAAGTACGACCAGCTTAAGAGCGAGAAAAAAATCAGCAACAATGCCGTGTTCATCGGCAGCTTCTACACCAGCGACATCATCGAGATCAACAACGAAAAGTTCCGGGTTATCGGAGTGAACAGCGACAAAAATAATCTGATCGAGGTGGACAGAATCGACATCAGACAGAAGGAATTCATCGAGCTGGAGGAAGAGAAGCAAGTGAATTACATGAACTCCGACTCATATCTCAAGAACAACCGGATCAAGGTGACCATCGGCAGAAAGACCACCAACATCGAGAAGTTTCACACCGACATCCTGGGAAACATGTACAAAAGCAAGCGGCCTAAGGCCCCTCAATTGGTGTTTAAAAAGGGC |
| CAS9 Amino Acid | MEKDYILGLDIGIGSVGYGLIDYDTKSIIDAGVRLFPEANADNNLGRRAKRGARRLKRRRIHRLERVKSLLSEYKIISGLAPTNNQPYNIRVKGLTEQLTKDELAVALLHIAKRRGIHNVDVAADKEETASDSLSTKDQINKNAKFLESRYVCELQKERLENEGHVRGVENRFLTKDIVREAKKIIDTQMQYYPEIDETFKEKYISLVETRREYYEGPGKGSPYGWDADVKKWYQLMMGHCTYFPVEFRSVKYAYTADLYNALNDLNNLTIARDDNPKLEYHEKYHIIENVFKQKRNPTLKQIAKEIGVNDINISGYRVTKSGKPQFTSFKLFHDLKKVVKDHAILDDIDLLNQIAEILTIYQDKDSIVAELGQLEYLMSEADKQSISELTGYTGTHSLSLKCMNMIIDELWHSSMNQMEVFTYLNMRPKKYELKGYQRIPTDMIDDAILSPVVKRSFKQAIGVVNAIIKKYGLPKDIIIELARESNSAEKSRYLRAIQKKNEKTRERIEAIIKEYGNENAKGLVQKIKLHDAQEGKCLYSLKDIPLEDLLRNPNNYDIDHIIPRSVSFDDSMHNKVLVRREQNAKKNNQTPYQYLTSGYADIKYSVFKQHVLNLAENKDRMTKKKREYLLEERNINKYDVQKEFINRNLVDTRYTTRELTTLLKTYFTINNLDVKVKTINGSFTDFLRKRWGFKKNRDEGYKHHAEDALIIANADYLFKEHKLLKEIKDVSDLAGDERNSNVKDEDQYEEVFGGYFKIEDIKKYKIKKFSHRVDKKPNRQLINDTIYSTRVKDDKRYLINTLKNLYDKSNGDLKERMQKDPESLLMYHHDPQTFEKLKIVMSQYENEKNPLAKYFEETGQYLTKYAKHDNGPAIHKIKYYGNKLVEHLDITKNYHNPQNKVVQLSQKSFRFDVYQTDKGYKFISIAYLTLKNEKNYYAISQEKYDQLKSEKKISNNAVFIGSFYTSDIIEINNEKFRVIGVNSDKNNLIEVDRIDIRQKEFIELEEEKQVNYMNSDSYLKNNRIKVTIGRKTTNIEKFHTDILGNMYKSKRPKAPQLVFKKG |
| Lengh(aa) | 1063 |
| repeat | GTTTTAGTACTCTGTAATTTTAGGTATGAATGATAC |
| tracrRNA | TGTATTTATACCTAAAATTACAGAATCTACTAAAACAAGACTATATGTCGTGTTTATCCCGTCCAATTGTGGTGGGATTTTTT |
| sgRNA | GTTTTAGTACTCTGgaaaCAGAATCTACTAAAACAAGACTATATGTCGTGTTTATCCCGTCCAATTGTGGTGGGATTTTTT |

| Cas9 ID in NCBI | WP_154836552 |
| --- | --- |
| Host strain | Staphylococcus haemolyticus |
| Nuclease name | Sha2Cas9 |
| Human codon-optimized Cas9_Gene | ATGACCACCAACTACATCCTGGGCCTGGATATCGGCATCACCAGCGTGGGCTACGGCATCATTAACTACGAAGATAAGACCATCATCGACGCCGGCGTGAGACTGTTCCCCGAGGCTAATGTGGAAAACAACGAAGGTAGAAGAAGCAAGCGGGGCGCCCGGAGACTGAAGCGGAGAAGAATCCACAGACTGGATAGAATCAAACAGCTGCTCAATGAATACAAGCTGGTTGAGCTGAACGACGTGCCGAAGAGCACAAACCCCTACGATATCAGAGTGAAGGGCCTGAAAGAGGAACTGACAAGAGAAGAGCTCGTGATCGCTCTGCTGCACCTGGCTAAGAGAAGAGGCATCCACAACATCGACGTGGTGGAGCAGGACGGCGAAGAGGGAAACCAGCTGAGCACCAAGGAGCAGCTGTCTAAGAATAAGAACCTGCTGAAGGATAAGTTCGTGTGCGAGCTTCTGCTGGAAAGATTCAACGAGGGCAAGGTGCGGGGCGAAGAGAATCGCTTCAAGACGAGCGACATCATCAAGGAAGCCGAGCAAATCCTGAAGGTTCAGAAGAATATCCATAATCTGGACGAGCACTTCATCAATAAATACATTGAGCTGGTGAAGACCCGCAGAGAATATTTTGAAGGCCCTGGCGAGGGCAGCCCTTTCGGCTGGAACGGCGACCTGAAAAAGTGGTACGAGATGCTGATGGGCCACTGCACCTACTTCCCTGAGGAACTGAGATCCGTGAAATACGCCTACTCGGCCGATCTCTTTAACGCCCTGAACGACTTAAACAACCTGGTGATCCAAAGAGATGGATCAACAAAGCTGGAATACTATGAAAAGTATCATATCATCGAGAACGTGTTCAAGCAAAAGAAAAAGCCCACCCTGAAACAGATCGCTAACGAGATCGGCGTCACCCCTGAGGACATCAAGGGCTACAGAATCACCAAGTCCGGCAAGGAAAACTTCACCGAATTCAAACTGTACCACGACCTGAAAAAAGTCCTGAAGGACCAGAGCATCCTGGAGAACGTGAGCCTGCTGGACCAGATCGCCGAGATCCTCACAGTGTACCAGGACAAAGGCTCGATCAAGAATGAGCTGAGCAAGCTGGACGAGACAATTAACGAAACCGACAAGGAAAACATCAGCAACCTGACCGGCTACAACGGCACACACCGGCTGAGCCTCAAGTGTATCAACCTGGTGTTGGAGGAACTGTGGCACAGCAGCAGAAACCAAATGGAGATCTTCTCTTATCTCAACATCAAACCTAAGAAGATCGACCTCAAAAAGAGCAACAAAATCCCTAAGGACATGATCGACGAGTTCATTCTGTCTCCTGTGGTGAAGCGGACATTCGGCCAGGCCATCAATGTGATCAACAAAATCATCGAGAAGTACGGAATCCCCAACGACATCATCATCGAGCTGGCCAGAGAGAACAACAGCAAGGACAAACAGAAGTTCATCAACGAACTGCAGCGCAAGAATGAGAAGACACGGCAGAGAATCAACGAGATCATCGGGGAATACGGCAACCAGAACGCCAAGCGGCTGGTTGAAAAGATTAAGCTGCACGACGAGCAGGAGGGAAAGTGCCTGTACAGCCTGGAAAGCATCCCACTGGAAGATTTGCTGAATAACCCCAACTACTACGAGGTGGACCACATCATCCCTAGATCTGTGAGCTTTGACAATAGCTATCAGAACAAAGTGCTGGTGAAACAGACAGAGAACAGCAAGAAGGGCAACAGAACACCTTACCAATATCTGAACAGCGGAGAGGCCAAAATCAGCTACAACCAGTTCAAGCAGCACATCCTGAATCTGAGCAAGTCCAAGGACCGGATCAGCAAGAAGAAGAAGGAGTACCTGCTGGAGGAGCGGGACATCAACAAGTTCGAAGTGAAGAAGGAATTCATAAATAGAAACCTGGTGGACACCCGGTACGCCACCAGAGAGCTTAGCAATTACCTGAAAGCCTACTTCAGCGCCAACGACATGAACGTGAAGATCAAGACCATCAACGGCTCTTTTACCGACTACCTGAGGAAGGTGTGGAAGTTCAAAAAGGAACGGAACCACGGCTACAAGCACCACGCCGAGGACGCCCTGATCATCGCCAATGCCGACTTCCTGTTTAAGGAGAATAAGAAGCTGAAGGAGGCTAACAAGGTGCTGGAGAAACCTACCGAAAGCAATGAACTGAACAAGGTCGAGAACTCTACACGGATCAAGAACGAAGATGAATACAAGGAACTGTTCAACATCCCCCAGCAGGTGACCGATATCAAGGAGTTCAAAGACTTCAAGTTCAGCCACCGGGTGGATAAAAAACCAAACAGACAGCTCATCAACGACACCCTGTACTCCACCAGACAGGTTGGAGATGATGCTTATATCGTGCAGACCATCAAGAATATCTATTCTAAAGATAACACCGACCTGAAGAAGCACTTCAACAAGAACCCTGAGAAATTTCTGATGTACCAGCATGATCCAAAGACCTTTGAGAAGCTGGAAACCATCATGAAGCAGTACTCCAACGAAAAGAACCCCCTGGCTAAATACCACGAGGAAACCGGCGAATACCTGACCAAGTACAGCAAGAAGGACAACGGCCCTATCGTGAAGACAATTAAAATGCTGTCCAACAAGGTCGGCAATCACCTGGACGTGACACACAAGTACGAGGACAGCAAGAAGCAGCTGGTGAGGCTGAGCATCAAGAGCCTGCGGTTCGACGTTTACCTGACCGACAAGGGCTACAAGTTCATTACTATCTCCTACCTGGATATTCTGAAGAAGGACAGCTACTACTACATTCCTACAGAGACATACAACAACTTAAAGGCCAACAAATCTATCGACAAAAGCAGCCAGTTCATCGGATCTTTCTACTATAACGATCTCATCGAGCTGGATGGCGAAGTGTACAAGATCATTGGAGTGAACTCTGATAAGAGAAACCTGATCGAGCTGGACCTGCCCAACATCAGATACAAGGAGTACTGCGAGCTGAATAACGTGAAGGGCGAAGCCAGAATCAAGAAGACCATTGGCAAGAAAGTCAAGTCCATCAAAATCCTGCACACCGACGTGCTGGGAAATATCTACTACCAGAAAAACATCCAGAAGCCTCAACTGCTGTTTAAGAGGGGCAAC |
| CAS9 Amino Acid | MTTNYILGLDIGITSVGYGIINYEDKTIIDAGVRLFPEANVENNEGRRSKRGARRLKRRRIHRLDRIKQLLNEYKLVELNDVPKSTNPYDIRVKGLKEELTREELVIALLHLAKRRGIHNIDVVEQDGEEGNQLSTKEQLSKNKNLLKDKFVCELLLERFNEGKVRGEENRFKTSDIIKEAEQILKVQKNIHNLDEHFINKYIELVKTRREYFEGPGEGSPFGWNGDLKKWYEMLMGHCTYFPEELRSVKYAYSADLFNALNDLNNLVIQRDGSTKLEYYEKYHIIENVFKQKKKPTLKQIANEIGVTPEDIKGYRITKSGKENFTEFKLYHDLKKVLKDQSILENVSLLDQIAEILTVYQDKGSIKNELSKLDETINETDKENISNLTGYNGTHRLSLKCINLVLEELWHSSRNQMEIFSYLNIKPKKIDLKKSNKIPKDMIDEFILSPVVKRTFGQAINVINKIIEKYGIPNDIIIELARENNSKDKQKFINELQRKNEKTRQRINEIIGEYGNQNAKRLVEKIKLHDEQEGKCLYSLESIPLEDLLNNPNYYEVDHIIPRSVSFDNSYQNKVLVKQTENSKKGNRTPYQYLNSGEAKISYNQFKQHILNLSKSKDRISKKKKEYLLEERDINKFEVKKEFINRNLVDTRYATRELSNYLKAYFSANDMNVKIKTINGSFTDYLRKVWKFKKERNHGYKHHAEDALIIANADFLFKENKKLKEANKVLEKPTESNELNKVENSTRIKNEDEYKELFNIPQQVTDIKEFKDFKFSHRVDKKPNRQLINDTLYSTRQVGDDAYIVQTIKNIYSKDNTDLKKHFNKNPEKFLMYQHDPKTFEKLETIMKQYSNEKNPLAKYHEETGEYLTKYSKKDNGPIVKTIKMLSNKVGNHLDVTHKYEDSKKQLVRLSIKSLRFDVYLTDKGYKFITISYLDILKKDSYYYIPTETYNNLKANKSIDKSSQFIGSFYYNDLIELDGEVYKIIGVNSDKRNLIELDLPNIRYKEYCELNNVKGEARIKKTIGKKVKSIKILHTDVLGNIYYQKNIQKPQLLFKRGN |
| Lengh(aa) | 1058 |
| repeat | GTTTTAGTACTCTGTAATTTTAGGTATAAGTGAGAC |
| tracrRNA | TTGTACTTATACCTAAAATTACAGAATCTACTAAAACAAGACTATATGTCGTGTTTATCCCAACAATTTATTGTTGGGATTTTTT |
| sgRNA | GTTTTAGTACTCTGgaaaCAGAATCTACTAAAACAAGACTATATGTCGTGTTTATCCCAACAATTTATTGTTGGGATTTTTT |

| Cas9 ID in NCBI | WP_115359133 |
| --- | --- |
| Host strain | Staphylococcus petrasii |
| Nuclease name | SpeCas9 |
| Human codon-optimized Cas9_Gene | ATGGCCACAAACTACATCTTGGGCCTGGATATCGGAATCACAAGCGTGGGATACGGCATCATCAACTATGAGGACAAGACCATCATCGACGCCGGCGTGCGGCTGTTTCCAGAGGCCAATGTGGAAAATAACGAAGGCAGAAGAAGCAAGAGAGGCGCCAGACGTTTAAAGCGGCGGCGGATCCACAGACTGGACCGGGTGAAGCAGCTGCTGAACGAGTACAAGCTGGTGAAGCTGAATGACATCCCCAAGTCCACCAACCCTTATGAGATCCGCGTGAAGGGCCTGAGAGAGGAACTGACACGGGATGAGCTGGTGATCGCCCTGCTGCACCTGGCCAAGCGGAGAGGAATCCACAATATCGAGGTTATCGAGCAGGACAACGAGGAAGGGAACCAGCTGAGCACAAAGGAGCAGTTGTCTAAGAACAACAACCTGCTGAAGGATAAGTTCGTGTGTGAACTGCTGCTGGAAAGATTTAACGACGGCAAGGTGCGGGGCGAGGAAAATAGGTTCAAGACCTCCGACATCATTAAGGAAGCTAAGCAGATTCTGGAAGTGCAAAAGGACGTGCACAACCTGGACGATTACTTCATCAACAAGTACATCGAGCTGGTCGAGACAAGACGGGAATATTACGAGGGCCCCGGAGAAGGCTCTCCTTTCGGCTGGGACGGCGACCTGAAGAAATGGTACGAGATGCTGATGGGCAGATGTACCTACTTTCCTGAGGAGCTGAGATCTGTGAAATACGCTTACAGCGCCGACCTGTTCAACGCCCTGAACGACCTGAACAATCTGGTGATCCAGCGGGACGGCTCTACGAAACTGGAATACCACGAGAAGTACCACATCATCGAAAACGTGTTCAAGCAGAAGAAGAAGCCTACACTGAAACAGATCGCTAACGAGATCGAGGTGACCCCTGAAGATATCAAGGGCTATAGAATCACAAAGAGCGGCAAGGAAAATTTCACAGAGTTCAAACTGTATCACGATCTGAAAAAGGTGCTGAAGGACCAGAGCATCCTGGAAAACGTTTCTCTGCTGGATCAGATCGCCGAAATCCTGACCGTGTACCAGGATAAAGAATCCATCAAGAAAGAGCTGTCCAAGCTGGACGAAACCATCAATGATATTGACAAGGAAAGCATCAGCAACCTGACAGGCTATAACGGCACCCACCGGCTGTCTCTGAAGTGCATCAACCTGGTCCTAGAAGAGCTGTGGCACAGCAGCAGAAATCAAATGGAAATTTTCAGCTACCTGAACATCAAGCCTAAAAAGATCGATCTGAAGAAGAGCAACAAGATCCCTAAGGACATGATCGACGAGTTCATCCTGAGCCCTGTGGTGAAGAGAACATTTGGCCAGGCCATCAACGTGATCAACAAGGTGATCGAGAAATACGGAGTGCCCAACGACATCATCATCGAACTGGCCAGAGAGAATAACAGCAAGGACAAGCAGAAATTCATCAACGAGCTTCAGAAGAAGAACGAGAAGACCCGGCAGAGAATCAATGAGATCATCGGAGAATACGGCAATCAGAACGCTAAGCGGCTGGTGGAAAAGATCAGACTGCACGACGAACAGGAGGGAAAGTGCCTGTACTCTCTGGAAAGCATCCCACTGGATGATCTTCTGAATAATCCAAATTACTACGAGGTGGACCACATCATCCCTAGAAGCGTGTCTTTCGACAACAGCTACCAAAACAAAGTGCTGGTGAAGCAGACCGAGAATAGCAAGAAAGGCAACAGAACCCCTTACCAGTACCTGAACTCCGGCGAGGCCAAGATCAGCTACAATCAGTTTAAGCAGCACATCCTGAACCTGTCAAAGTCTAAGGACAGAATCTCAAAGAAGAAGAAGGAATACCTGCTGGAAGAGCGGGACATTAACAAGTTCGAGGTGAAGAAGGAATTCATCAACAGAAACCTCGTTGATACCAGATACGCCACCAGAGAGCTGAGCAACTACCTGAAGGCCTACTTCTCCGCCAACGACATGAACGTAAAGATCAAGACAATCAACGGCAGCTTCACCGACTACCTCAGAAAGGTGTGGAAGTTCAAGAAGGAACGGAACCACGGCTACAAGCACCACGCCGAGGACGCACTGATCATCGCTAATGCCGACTTCCTGTTCAAGGAAAACAAAAAACTCAAGGAAGTGAACAAAGTGCTAGAGAAGCCCGCCGACAATAACGAACTTAATAAAGTCGAGAACTCTATCGGCATCAAAACCGAGGATGAGTACAAGGAGCTGTTTAACATCCCCCAGCAGGTGGCCGACATCAAAGAGTTCAAGGACTTCAAGTTTAGCCACCGCGTGGATAAGAAACCAAACCGGCAACTAATCAACGACACCCTGTACAGCACCAGACAGCTGGGCGAGGACAGCTACATCGTGCAGACCATCAAAAACATTTACAGCAAGGACAACACCGATCTGAAGAAGCATTTCAACAAAAATCCTGAGAAGTTCCTGATGTACCAGCATGATCCCAAGACCTTCGAGAAGCTGGAAACCATCATGAAACAATACAGCAATGAAAAGAACCCCCTGGCCAAGTACCATGAGGAAACCGGCGAGTACCTGACCAAGTATAGTAAGAAGAACAACGGCCCTATCGTGAAGACAATCAAGATGCTGGGCAACAAGGTGGGCAATCACCTGGACGTGACCCACAAGTACAAGAACAGCAATAAGCAACTGGTGAGGCTGTCCATAAAGTCCCTCAGATTTGACGTGTACCTTACAGACAAGGGATACAAGTTCATCACCATCTCTTACCTGGACATCCTGAAAAAAGACAGCTACTACTACATCCCTATTGAAACCTACGAGAAGCTGAAAACAAACAAGGGCATCAACAAGTCGAGCCAGTTCATCGGCAGCTTCTACTACAACGACCTGATCGAGCTGGATGGCGAAGTGTACAAGATCATAGGTGTGAACAGCGACAAGCGCAACATCATTGAGCTGGACCTGCCTAACATCAGATACAAGGAATACTGCGAGCTGAACAACGTGAAGGGAGAAGCCAGAATCAAGAAGTCCATAGGCAAGAAGGTGCAGAGCATCAAAAAGCTGAGCACCGACGTGCTGGGAAACAGATACTATCAGAAGAACGTCCAGAACCCTCAGCTGCTGTTTAAACGGGGCAAC |
| CAS9 Amino Acid | MATNYILGLDIGITSVGYGIINYEDKTIIDAGVRLFPEANVENNEGRRSKRGARRLKRRRIHRLDRVKQLLNEYKLVKLNDIPKSTNPYEIRVKGLREELTRDELVIALLHLAKRRGIHNIEVIEQDNEEGNQLSTKEQLSKNNNLLKDKFVCELLLERFNDGKVRGEENRFKTSDIIKEAKQILEVQKDVHNLDDYFINKYIELVETRREYYEGPGEGSPFGWDGDLKKWYEMLMGRCTYFPEELRSVKYAYSADLFNALNDLNNLVIQRDGSTKLEYHEKYHIIENVFKQKKKPTLKQIANEIEVTPEDIKGYRITKSGKENFTEFKLYHDLKKVLKDQSILENVSLLDQIAEILTVYQDKESIKKELSKLDETINDIDKESISNLTGYNGTHRLSLKCINLVLEELWHSSRNQMEIFSYLNIKPKKIDLKKSNKIPKDMIDEFILSPVVKRTFGQAINVINKVIEKYGVPNDIIIELARENNSKDKQKFINELQKKNEKTRQRINEIIGEYGNQNAKRLVEKIRLHDEQEGKCLYSLESIPLDDLLNNPNYYEVDHIIPRSVSFDNSYQNKVLVKQTENSKKGNRTPYQYLNSGEAKISYNQFKQHILNLSKSKDRISKKKKEYLLEERDINKFEVKKEFINRNLVDTRYATRELSNYLKAYFSANDMNVKIKTINGSFTDYLRKVWKFKKERNHGYKHHAEDALIIANADFLFKENKKLKEVNKVLEKPADNNELNKVENSIGIKTEDEYKELFNIPQQVADIKEFKDFKFSHRVDKKPNRQLINDTLYSTRQLGEDSYIVQTIKNIYSKDNTDLKKHFNKNPEKFLMYQHDPKTFEKLETIMKQYSNEKNPLAKYHEETGEYLTKYSKKNNGPIVKTIKMLGNKVGNHLDVTHKYKNSNKQLVRLSIKSLRFDVYLTDKGYKFITISYLDILKKDSYYYIPIETYEKLKTNKGINKSSQFIGSFYYNDLIELDGEVYKIIGVNSDKRNIIELDLPNIRYKEYCELNNVKGEARIKKSIGKKVQSIKKLSTDVLGNRYYQKNVQNPQLLFKRGN |
| Lengh(aa) | 1058 |
| repeat | GTTTTAGTACTCTGTAATTTTAGGTATAAGTGAAAC |
| tracrRNA | GTACTTATACCTAAAATTACAGAATCTACTAAAACAAGACTATATGTCGTGTTTATCCCAACAATTTATTGTTGGGATTTTTT |
| sgRNA | GTTTTAGTACTCTGgaaaCAGAATCTACTAAAACAAGACTATATGTCGTGTTTATCCCAACAATTTATTGTTGGGATTTTTT |

| Cas9 ID in NCBI | WP_114599540 |
| --- | --- |
| Host strain | Staphylococcus warneri |
| Nuclease name | Swa2Cas9 |
| Human codon-optimized Cas9_Gene | ATGAAAGAAAAGTACATCCTGGGCCTGGACCTGGGCATCACAAGCGTGGGCTACGGGATCATCAACTTCGAAACCAAGAAGATCATCGATGCCGGCGTGCGGCTGTTCCCCGAGGCTAACGTAGACAACAATGAGGGCAGAAGAAGCAAGAGAGGCAGCCGGAGACTCAAGAGAAGACGGATCCACAGACTGGAGCGGGTGAAACTGCTGCTGACCGAGTACGACCTGATCAACAAAGAACAGATTCCTACAAGCAACAACCCCTACCAGATCAGAGTGAAGGGCCTGTCTGAAATCCTGTCCAAGGATGAGCTGGCCATCGCACTGCTCCATCTGGCCAAGCGGCGGGGCATCCATAACATCAATGTGTCCAGCGAGGATGAAGACGCTTCTAATGAACTGAGCACAAAGGAACAGATCAACCGGAACAACAAGCTGCTGAAGGACAAGTACGTGTGCGAGGTGCAGCTGCAGCGGCTGAAGGAAGGCCAGATCAGAGGAGAGAAAAATAGGTTCAAGACCACAGACATCCTGAAGGAGATCGACCAGCTGCTGAAAGTGCAAAAAGATTACCACAACCTGGACATCGACTTCATCAATCAATACAAGGAGATCGTGGAAACCCGCAGAGAATACTTCGAGGGACCAGGCCAGGGCAGCCCTTTCGGCTGGAACGGCGATCTGAAGAAATGGTACGAGATGCTGATGGGCCACTGCACCTACTTCCCTCAGGAGCTGAGAAGCGTGAAGTACGCCTACAGTGCCGATCTGTTTAACGCCCTGAATGATCTGAATAACCTCATCATTCAGAGAGATAACAGCGAGAAACTTGAATACCACGAGAAGTACCATATCATCGAGAACGTGTTCAAGCAGAAGAAGAAGCCTACCCTGAAACAGATCGCCAAAGAAATTGGCGTGAACCCCGAGGACATCAAAGGTTACAGAATCACCAAGTCCGGCACCCCTCAGTTTACAGAGTTCAAGCTGTATCACGACCTGAAGAGCATCGTGTTTGACAAGAGCATCCTGGAGAACGAGGCCATCCTGGATCAGATCGCTGAGATCCTGACCATCTACCAGGACGAGCAAAGCATCAAGGAGGAACTGAACAAGCTGCCTGAGATCCTGAACGAACAAGACAAGGCCGAGATCGCGAAGCTGATCGGCTATAACGGAACACACAGATTGTCCCTGAAGTGCATCCACCTGATCAACGAAGAGCTGTGGCAGACCAGCAGAAATCAGATGGAAATATTCAACTACCTGAACATCAAACCTAACAAGGTGGACCTGTCCGAGCAGAACAAGATCCCAAAGGACATGGTGAATGATTTTATCCTGTCTCCTGTGGTGAAGCGGACCTTCATTCAGTCCATAAATGTGATCAACAAGGTGATCGAGAAATACGGCATCCCTGAGGACATCATTATCGAACTGGCCAGAGAGAACAACAGCGACGATAGAAAGAAATTCATCAACAACCTGCAGAAAAAAAACGAGGCCACAAGAAAGAGAATTAACGAGATCATCGGCCAGACCGGAAACCAGAATGCCAAAAGAATCGTGGAAAAAATCCGGCTTCACGACCAGCAGGAGGGAAAGTGCCTGTACTCTCTGGAAAGCATCGCCCTGATGGACCTGCTGAACAATCCTCAAAACTACGAGGTGGACCACATCATCCCCAGGTCTGTGGCCTTCGATAACTCTATCCACAACAAGGTGCTGGTGAAGCAAATCGAGAACTCTAAAAAAGGCAACCGTACCCCATACCAGTACCTGAACAGCAGCGATGCTAAGCTGTCTTACAACCAATTTAAGCAGCACATCCTGAATCTGAGCAAGTCTAAAGATAGAATCAGCAAAAAAAAAAAGGACTACTTACTGGAGGAGAGAGACATCAACAAGTTCGAGGTGCAAAAAGAATTCATCAACCGGAATCTGGTTGATACAAGATACGCCACCAGAGAGCTCACCTCTTACCTGAAGGCCTATTTTAGCGCCAATAACATGGATGTGAAGGTCAAGACAATTAATGGCAGCTTCACCAACCACCTGCGGAAAGTGTGGCGGTTCGACAAGTACAGAAACCACGGCTACAAGCACCACGCCGAGGATGCCCTGATCATCGCCAACGCCGACTTCCTGTTCAAGGAGAATAAAAAACTCCAGAACGCTAATAAGATCCTGGAAAAGCCAACCATCGAAAACAACACTAAGAAGGTGACCGTGGAAAAGGAAGAAGACTACAACAACATGTTCGAGACACCTAAGCTGGTGGAAGACATTAAGCAGTACAGAGATTACAAGTTCAGCCACAGAGTGGACAAGAAGCCCAACCGCCAGCTGATAAACGACACCCTGTACAGCACAAGAATGAAGGACGAGCACGATTACATTGTCCAGACCATCACAGATATCTACGGCAAGGACAACACCAACCTCAAGAAGCAGTTCAACAAGAACCCCGAGAAGTTTCTGATGTACCAGAACGACCCCAAGACCTTCGAGAAGCTCAGCATCATCATGAAGCAGTATAGTGACGAGAAGAACCCTCTGGCTAAATATTATGAAGAGACAGGCGAGTACCTGACCAAGTACAGCAAGAAGAACAACGGCCCTATCGTGAAGAAGATCAAGCTGCTCGGCAACAAAGTTGGAAATCACCTGGACGTGACAAACAAGTACGAGAACAGCACCAAGAAGCTGGTCAAACTCAGCATCAAGAACTACAGATTCGACGTGTACCTGACAGAAAAGGGCTACAAGTTCGTGACCATCGCTTATCTGAACGTGTTCAAAAAGGATAACTACTACTACATCCCTAAGGACAAGTACCAGGAGCTGAAGGAAAAAAAGAAGATCAAGGACACAGACCAGTTCATCGCCTCTTTTTACAAGAATGACCTGATCAAGCTGAACGGCGACCTGTACAAGATCATCGGAGTGAACAGCGACGACAGAAACATCATCGAACTGGACTATTACGATATTAAGTACAAGGATTATTGTGAAATCAATAACATCAAGGGAGAACCCAGAATCAAGAAGACCATCGGCAAGAAGACCGAAAGCATCGAGAAATTCACCACGGACGTGCTGGGTAACCTGTACCTGCACAGCACCGAGAAAGCCCCTCAACTGATCTTCAAGCGGGGCCTG |
| CAS9 Amino Acid | MKEKYILGLDLGITSVGYGIINFETKKIIDAGVRLFPEANVDNNEGRRSKRGSRRLKRRRIHRLERVKLLLTEYDLINKEQIPTSNNPYQIRVKGLSEILSKDELAIALLHLAKRRGIHNINVSSEDEDASNELSTKEQINRNNKLLKDKYVCEVQLQRLKEGQIRGEKNRFKTTDILKEIDQLLKVQKDYHNLDIDFINQYKEIVETRREYFEGPGQGSPFGWNGDLKKWYEMLMGHCTYFPQELRSVKYAYSADLFNALNDLNNLIIQRDNSEKLEYHEKYHIIENVFKQKKKPTLKQIAKEIGVNPEDIKGYRITKSGTPQFTEFKLYHDLKSIVFDKSILENEAILDQIAEILTIYQDEQSIKEELNKLPEILNEQDKAEIAKLIGYNGTHRLSLKCIHLINEELWQTSRNQMEIFNYLNIKPNKVDLSEQNKIPKDMVNDFILSPVVKRTFIQSINVINKVIEKYGIPEDIIIELARENNSDDRKKFINNLQKKNEATRKRINEIIGQTGNQNAKRIVEKIRLHDQQEGKCLYSLESIALMDLLNNPQNYEVDHIIPRSVAFDNSIHNKVLVKQIENSKKGNRTPYQYLNSSDAKLSYNQFKQHILNLSKSKDRISKKKKDYLLEERDINKFEVQKEFINRNLVDTRYATRELTSYLKAYFSANNMDVKVKTINGSFTNHLRKVWRFDKYRNHGYKHHAEDALIIANADFLFKENKKLQNANKILEKPTIENNTKKVTVEKEEDYNNMFETPKLVEDIKQYRDYKFSHRVDKKPNRQLINDTLYSTRMKDEHDYIVQTITDIYGKDNTNLKKQFNKNPEKFLMYQNDPKTFEKLSIIMKQYSDEKNPLAKYYEETGEYLTKYSKKNNGPIVKKIKLLGNKVGNHLDVTNKYENSTKKLVKLSIKNYRFDVYLTEKGYKFVTIAYLNVFKKDNYYYIPKDKYQELKEKKKIKDTDQFIASFYKNDLIKLNGDLYKIIGVNSDDRNIIELDYYDIKYKDYCEINNIKGEPRIKKTIGKKTESIEKFTTDVLGNLYLHSTEKAPQLIFKRGL |
| Lengh(aa) | 1054 |
| repeat | GTTTTAGTACTCTGTAATTTTAGGTATGAGTGAAAC |
| tracrRNA | TTGTACTCATACCTAAAATTACAGAATCTACTGAAACAAGACTATATGTCGTGTTTATCCCACTAATTTATTAGTGGGATTTTTT |
| sgRNA | GTTTTAGTACTCTGgaaaCAGAATCTACTGAAACAAGACTATATGTCGTGTTTATCCCACTAATTTATTAGTGGGATTTTTT |

| Cas9 ID in NCBI | WP_154836552 |
| --- | --- |
| Host strain | Staphylococcus haemolyticus |
| Nuclease name | Sha2Cas9-HF |
| Human codon-optimized Cas9_Gene | ATGACCACCAACTACATCCTGGGCCTGGATATCGGCATCACCAGCGTGGGCTACGGCATCATTAACTACGAAGATAAGACCATCATCGACGCCGGCGTGAGACTGTTCCCCGAGGCTAATGTGGAAAACAACGAAGGTAGAAGAAGCAAGCGGGGCGCCCGGAGACTGAAGCGGAGAAGAATCCACAGACTGGATAGAATCAAACAGCTGCTCAATGAATACAAGCTGGTTGAGCTGAACGACGTGCCGAAGAGCACAAACCCCTACGATATCAGAGTGAAGGGCCTGAAAGAGGAACTGACAAGAGAAGAGCTCGTGATCGCTCTGCTGCACCTGGCTAAGAGAAGAGGCATCCACAACATCGACGTGGTGGAGCAGGACGGCGAAGAGGGAAACCAGCTGAGCACCAAGGAGCAGCTGTCTAAGAATAAGAACCTGCTGAAGGATAAGTTCGTGTGCGAGCTTCTGCTGGAAAGATTCAACGAGGGCAAGGTGCGGGGCGAAGAGAATCGCTTCAAGACGAGCGACATCATCAAGGAAGCCGAGCAAATCCTGAAGGTTCAGAAGAATATCCATAATCTGGACGAGCACTTCATCAATAAATACATTGAGCTGGTGAAGACCCGCAGAGAATATTTTGAAGGCCCTGGCGAGGGCAGCCCTTTCGGCTGGAACGGCGACCTGAAAAAGTGGTACGAGATGCTGATGGGCCACTGCACCTACTTCCCTGAGGAACTGGCCTCCGTGAAATACGCCTACTCGGCCGATCTCTTTAACGCCCTGAACGACTTAAACAACCTGGTGATCCAAAGAGATGGATCAACAAAGCTGGAATACTATGAAAAGTATCATATCATCGAGAACGTGTTCAAGCAAAAGAAAAAGCCCACCCTGAAACAGATCGCTAACGAGATCGGCGTCACCCCTGAGGACATCAAGGGCTACAGAATCACCAAGTCCGGCAAGGAAAACTTCACCGAATTCAAACTGTACCACGACCTGAAAAAAGTCCTGAAGGACCAGAGCATCCTGGAGAACGTGAGCCTGCTGGACCAGATCGCCGAGATCCTCACAGTGTACCAGGACAAAGGCTCGATCAAGAATGAGCTGAGCAAGCTGGACGAGACAATTAACGAAACCGACAAGGAAAACATCAGCAACCTGACCGGCTACAACGGCACACACCGGCTGAGCCTCAAGTGTATCAACCTGGTGTTGGAGGAACTGTGGCACAGCAGCAGAGCCCAAATGGAGATCTTCTCTTATCTCAACATCAAACCTAAGAAGATCGACCTCAAAAAGAGCAACAAAATCCCTAAGGACATGATCGACGAGTTCATTCTGTCTCCTGTGGTGAAGCGGACATTCGGCCAGGCCATCAATGTGATCAACAAAATCATCGAGAAGTACGGAATCCCCAACGACATCATCATCGAGCTGGCCAGAGAGAACAACAGCAAGGACAAACAGAAGTTCATCAACGAACTGCAGCGCAAGAATGAGAAGACACGGCAGAGAATCAACGAGATCATCGGGGAATACGGCAACCAGAACGCCAAGCGGCTGGTTGAAAAGATTAAGCTGCACGACGAGCAGGAGGGAAAGTGCCTGTACAGCCTGGAAAGCATCCCACTGGAAGATTTGCTGAATAACCCCAACTACTACGAGGTGGACCACATCATCCCTAGATCTGTGAGCTTTGACAATAGCTATCAGAACAAAGTGCTGGTGAAACAGACAGAGAACAGCAAGAAGGGCAACAGAACACCTTACCAATATCTGAACAGCGGAGAGGCCAAAATCAGCTACAACCAGTTCAAGCAGCACATCCTGAATCTGAGCAAGTCCAAGGACCGGATCAGCAAGAAGAAGAAGGAGTACCTGCTGGAGGAGCGGGACATCAACAAGTTCGAAGTGAAGAAGGAATTCATAAATAGAAACCTGGTGGACACCCGGTACGCCACCAGAGAGCTTAGCAATTACCTGAAAGCCTACTTCAGCGCCAACGACATGAACGTGAAGATCAAGACCATCAACGGCTCTTTTACCGACTACCTGAGGAAGGTGTGGAAGTTCAAAAAGGAACGGAACCACGGCTACAAGCACCACGCCGAGGACGCCCTGATCATCGCCAATGCCGACTTCCTGTTTAAGGAGAATAAGAAGCTGAAGGAGGCTAACAAGGTGCTGGAGAAACCTACCGAAAGCAATGAACTGAACAAGGTCGAGAACTCTACACGGATCAAGAACGAAGATGAATACAAGGAACTGTTCAACATCCCCCAGCAGGTGACCGATATCAAGGAGTTCAAAGACTTCAAGTTCAGCCACCGGGTGGATAAAAAACCAAACAGACAGCTCATCAACGACACCCTGTACTCCACCAGACAGGTTGGAGATGATGCTTATATCGTGCAGACCATCAAGAATATCTATTCTAAAGATAACACCGACCTGAAGAAGCACTTCAACAAGAACCCTGAGAAATTTCTGATGTACCAGCATGATCCAAAGACCTTTGAGAAGCTGGAAACCATCATGAAGCAGTACTCCAACGAAAAGAACCCCCTGGCTAAATACCACGAGGAAACCGGCGAATACCTGACCAAGTACAGCAAGAAGGACAACGGCCCTATCGTGAAGACAATTAAAATGCTGTCCAACAAGGTCGGCAATCACCTGGACGTGACACACAAGTACGAGGACAGCAAGAAGCAGCTGGTGAGGCTGAGCATCAAGAGCCTGCGGTTCGACGTTTACCTGACCGACAAGGGCTACAAGTTCATTACTATCTCCTACCTGGATATTCTGAAGAAGGACAGCTACTACTACATTCCTACAGAGACATACAACAACTTAAAGGCCAACAAATCTATCGACAAAAGCAGCCAGTTCATCGGATCTTTCTACTATAACGATCTCATCGAGCTGGATGGCGAAGTGTACAAGATCATTGGAGTGAACTCTGATAAGAGAAACCTGATCGAGCTGGACCTGCCCAACATCAGATACAAGGAGTACTGCGAGCTGAATAACGTGAAGGGCGAAGCCAGAATCAAGAAGACCATTGGCAAGAAAGTCAAGTCCATCAAAATCCTGCACACCGACGTGCTGGGAAATATCTACTACCAGAAAAACATCCAGAAGCCTCAACTGCTGTTTAAGAGGGGCAAC |
| CAS9 Amino Acid | MTTNYILGLDIGITSVGYGIINYEDKTIIDAGVRLFPEANVENNEGRRSKRGARRLKRRRIHRLDRIKQLLNEYKLVELNDVPKSTNPYDIRVKGLKEELTREELVIALLHLAKRRGIHNIDVVEQDGEEGNQLSTKEQLSKNKNLLKDKFVCELLLERFNEGKVRGEENRFKTSDIIKEAEQILKVQKNIHNLDEHFINKYIELVKTRREYFEGPGEGSPFGWNGDLKKWYEMLMGHCTYFPEELASVKYAYSADLFNALNDLNNLVIQRDGSTKLEYYEKYHIIENVFKQKKKPTLKQIANEIGVTPEDIKGYRITKSGKENFTEFKLYHDLKKVLKDQSILENVSLLDQIAEILTVYQDKGSIKNELSKLDETINETDKENISNLTGYNGTHRLSLKCINLVLEELWHSSANQMEIFSYLNIKPKKIDLKKSNKIPKDMIDEFILSPVVKRTFGQAINVINKIIEKYGIPNDIIIELARENNSKDKQKFINELQRKNEKTRQRINEIIGEYGNQNAKRLVEKIKLHDEQEGKCLYSLESIPLEDLLNNPNYYEVDHIIPRSVSFDNSYQNKVLVKQTENSKKGNRTPYQYLNSGEAKISYNQFKQHILNLSKSKDRISKKKKEYLLEERDINKFEVKKEFINRNLVDTRYATRELSNYLKAYFSANDMNVKIKTINGSFTDYLRKVWKFKKERNHGYKHHAEDALIIANADFLFKENKKLKEANKVLEKPTESNELNKVENSTRIKNEDEYKELFNIPQQVTDIKEFKDFKFSHRVDKKPNRQLINDTLYSTRQVGDDAYIVQTIKNIYSKDNTDLKKHFNKNPEKFLMYQHDPKTFEKLETIMKQYSNEKNPLAKYHEETGEYLTKYSKKDNGPIVKTIKMLSNKVGNHLDVTHKYEDSKKQLVRLSIKSLRFDVYLTDKGYKFITISYLDILKKDSYYYIPTETYNNLKANKSIDKSSQFIGSFYYNDLIELDGEVYKIIGVNSDKRNLIELDLPNIRYKEYCELNNVKGEARIKKTIGKKVKSIKILHTDVLGNIYYQKNIQKPQLLFKRGN |
| Lengh(aa) | 1058 |
| repeat | GTTTTAGTACTCTGTAATTTTAGGTATAAGTGAGAC |
| tracrRNA | TTGTACTTATACCTAAAATTACAGAATCTACTAAAACAAGACTATATGTCGTGTTTATCCCAACAATTTATTGTTGGGATTTTTT |
| sgRNA | GTTTTAGTACTCTGgaaaCAGAATCTACTAAAACAAGACTATATGTCGTGTTTATCCCAACAATTTATTGTTGGGATTTTTT |

| Cas9 ID in NCBI | WP_115359133 |
| --- | --- |
| Host strain | Staphylococcus petrasii |
| Nuclease name | SpeCas9-HF |
| Human codon-optimized Cas9_Gene | ATGGCCACAAACTACATCTTGGGCCTGGATATCGGAATCACAAGCGTGGGATACGGCATCATCAACTATGAGGACAAGACCATCATCGACGCCGGCGTGCGGCTGTTTCCAGAGGCCAATGTGGAAAATAACGAAGGCAGAAGAAGCAAGAGAGGCGCCAGACGTTTAAAGCGGCGGCGGATCCACAGACTGGACCGGGTGAAGCAGCTGCTGAACGAGTACAAGCTGGTGAAGCTGAATGACATCCCCAAGTCCACCAACCCTTATGAGATCCGCGTGAAGGGCCTGAGAGAGGAACTGACACGGGATGAGCTGGTGATCGCCCTGCTGCACCTGGCCAAGCGGAGAGGAATCCACAATATCGAGGTTATCGAGCAGGACAACGAGGAAGGGAACCAGCTGAGCACAAAGGAGCAGTTGTCTAAGAACAACAACCTGCTGAAGGATAAGTTCGTGTGTGAACTGCTGCTGGAAAGATTTAACGACGGCAAGGTGCGGGGCGAGGAAAATAGGTTCAAGACCTCCGACATCATTAAGGAAGCTAAGCAGATTCTGGAAGTGCAAAAGGACGTGCACAACCTGGACGATTACTTCATCAACAAGTACATCGAGCTGGTCGAGACAAGACGGGAATATTACGAGGGCCCCGGAGAAGGCTCTCCTTTCGGCTGGGACGGCGACCTGAAGAAATGGTACGAGATGCTGATGGGCAGATGTACCTACTTTCCTGAGGAGCTGGCCTCTGTGAAATACGCTTACAGCGCCGACCTGTTCAACGCCCTGAACGACCTGAACAATCTGGTGATCCAGCGGGACGGCTCTACGAAACTGGAATACCACGAGAAGTACCACATCATCGAAAACGTGTTCAAGCAGAAGAAGAAGCCTACACTGAAACAGATCGCTAACGAGATCGAGGTGACCCCTGAAGATATCAAGGGCTATAGAATCACAAAGAGCGGCAAGGAAAATTTCACAGAGTTCAAACTGTATCACGATCTGAAAAAGGTGCTGAAGGACCAGAGCATCCTGGAAAACGTTTCTCTGCTGGATCAGATCGCCGAAATCCTGACCGTGTACCAGGATAAAGAATCCATCAAGAAAGAGCTGTCCAAGCTGGACGAAACCATCAATGATATTGACAAGGAAAGCATCAGCAACCTGACAGGCTATAACGGCACCCACCGGCTGTCTCTGAAGTGCATCAACCTGGTCCTAGAAGAGCTGTGGCACAGCAGCAGAGCCCAAATGGAAATTTTCGCCTACCTGAACATCAAGCCTAAAAAGATCGATCTGAAGAAGAGCAACAAGATCCCTAAGGACATGATCGACGAGTTCATCCTGAGCCCTGTGGTGAAGAGAACATTTGGCCAGGCCATCAACGTGATCAACAAGGTGATCGAGAAATACGGAGTGCCCAACGACATCATCATCGAACTGGCCAGAGAGAATAACAGCAAGGACAAGCAGAAATTCATCAACGAGCTTCAGAAGAAGAACGAGAAGACCCGGCAGAGAATCAATGAGATCATCGGAGAATACGGCAATCAGAACGCTAAGCGGCTGGTGGAAAAGATCAGACTGCACGACGAACAGGAGGGAAAGTGCCTGTACTCTCTGGAAAGCATCCCACTGGATGATCTTCTGAATAATCCAAATTACTACGAGGTGGACCACATCATCCCTAGAAGCGTGTCTTTCGACAACAGCTACCAAAACAAAGTGCTGGTGAAGCAGACCGAGAATAGCAAGAAAGGCAACAGAACCCCTTACCAGTACCTGAACTCCGGCGAGGCCAAGATCAGCTACAATCAGTTTAAGCAGCACATCCTGAACCTGTCAAAGTCTAAGGACAGAATCTCAAAGAAGAAGAAGGAATACCTGCTGGAAGAGCGGGACATTAACAAGTTCGAGGTGAAGAAGGAATTCATCAACAGAAACCTCGTTGATACCAGATACGCCACCAGAGAGCTGAGCAACTACCTGAAGGCCTACTTCTCCGCCAACGACATGAACGTAAAGATCAAGACAATCAACGGCAGCTTCACCGACTACCTCAGAAAGGTGTGGAAGTTCAAGAAGGAACGGAACCACGGCTACAAGCACCACGCCGAGGACGCACTGATCATCGCTAATGCCGACTTCCTGTTCAAGGAAAACAAAAAACTCAAGGAAGTGAACAAAGTGCTAGAGAAGCCCGCCGACAATAACGAACTTAATAAAGTCGAGAACTCTATCGGCATCAAAACCGAGGATGAGTACAAGGAGCTGTTTAACATCCCCCAGCAGGTGGCCGACATCAAAGAGTTCAAGGACTTCAAGTTTAGCCACCGCGTGGATAAGAAACCAAACCGGCAACTAATCAACGACACCCTGTACAGCACCAGACAGCTGGGCGAGGACAGCTACATCGTGCAGACCATCAAAAACATTTACAGCAAGGACAACACCGATCTGAAGAAGCATTTCAACAAAAATCCTGAGAAGTTCCTGATGTACCAGCATGATCCCAAGACCTTCGAGAAGCTGGAAACCATCATGAAACAATACAGCAATGAAAAGAACCCCCTGGCCAAGTACCATGAGGAAACCGGCGAGTACCTGACCAAGTATAGTAAGAAGAACAACGGCCCTATCGTGAAGACAATCAAGATGCTGGGCAACAAGGTGGGCAATCACCTGGACGTGACCCACAAGTACAAGAACAGCAATAAGCAACTGGTGAGGCTGTCCATAAAGTCCCTCAGATTTGACGTGTACCTTACAGACAAGGGATACAAGTTCATCACCATCTCTTACCTGGACATCCTGAAAAAAGACAGCTACTACTACATCCCTATTGAAACCTACGAGAAGCTGAAAACAAACAAGGGCATCAACAAGTCGAGCCAGTTCATCGGCAGCTTCTACTACAACGACCTGATCGAGCTGGATGGCGAAGTGTACAAGATCATAGGTGTGAACAGCGACAAGCGCAACATCATTGAGCTGGACCTGCCTAACATCAGATACAAGGAATACTGCGAGCTGAACAACGTGAAGGGAGAAGCCAGAATCAAGAAGTCCATAGGCAAGAAGGTGCAGAGCATCAAAAAGCTGAGCACCGACGTGCTGGGAAACAGATACTATCAGAAGAACGTCCAGAACCCTCAGCTGCTGTTTAAACGGGGCAAC |
| CAS9 Amino Acid | MATNYILGLDIGITSVGYGIINYEDKTIIDAGVRLFPEANVENNEGRRSKRGARRLKRRRIHRLDRVKQLLNEYKLVKLNDIPKSTNPYEIRVKGLREELTRDELVIALLHLAKRRGIHNIEVIEQDNEEGNQLSTKEQLSKNNNLLKDKFVCELLLERFNDGKVRGEENRFKTSDIIKEAKQILEVQKDVHNLDDYFINKYIELVETRREYYEGPGEGSPFGWDGDLKKWYEMLMGRCTYFPEELASVKYAYSADLFNALNDLNNLVIQRDGSTKLEYHEKYHIIENVFKQKKKPTLKQIANEIEVTPEDIKGYRITKSGKENFTEFKLYHDLKKVLKDQSILENVSLLDQIAEILTVYQDKESIKKELSKLDETINDIDKESISNLTGYNGTHRLSLKCINLVLEELWHSSANQMEIFAYLNIKPKKIDLKKSNKIPKDMIDEFILSPVVKRTFGQAINVINKVIEKYGVPNDIIIELARENNSKDKQKFINELQKKNEKTRQRINEIIGEYGNQNAKRLVEKIRLHDEQEGKCLYSLESIPLDDLLNNPNYYEVDHIIPRSVSFDNSYQNKVLVKQTENSKKGNRTPYQYLNSGEAKISYNQFKQHILNLSKSKDRISKKKKEYLLEERDINKFEVKKEFINRNLVDTRYATRELSNYLKAYFSANDMNVKIKTINGSFTDYLRKVWKFKKERNHGYKHHAEDALIIANADFLFKENKKLKEVNKVLEKPADNNELNKVENSIGIKTEDEYKELFNIPQQVADIKEFKDFKFSHRVDKKPNRQLINDTLYSTRQLGEDSYIVQTIKNIYSKDNTDLKKHFNKNPEKFLMYQHDPKTFEKLETIMKQYSNEKNPLAKYHEETGEYLTKYSKKNNGPIVKTIKMLGNKVGNHLDVTHKYKNSNKQLVRLSIKSLRFDVYLTDKGYKFITISYLDILKKDSYYYIPIETYEKLKTNKGINKSSQFIGSFYYNDLIELDGEVYKIIGVNSDKRNIIELDLPNIRYKEYCELNNVKGEARIKKSIGKKVQSIKKLSTDVLGNRYYQKNVQNPQLLFKRGN |
| Lengh(aa) | 1058 |
| repeat | GTTTTAGTACTCTGTAATTTTAGGTATAAGTGAAAC |
| tracrRNA | GTACTTATACCTAAAATTACAGAATCTACTAAAACAAGACTATATGTCGTGTTTATCCCAACAATTTATTGTTGGGATTTTTT |
| sgRNA | GTTTTAGTACTCTGgaaaCAGAATCTACTAAAACAAGACTATATGTCGTGTTTATCCCAACAATTTATTGTTGGGATTTTTT |
