# Supplementary material for: Identification of SaCas9 orthologs containing a conserved serine residue that determines simple NNGG PAM recognition
Source: PLoS Biol. 2022 Nov 30;20(11):e3001897. doi: 10.1371/journal.pbio.3001897 (PMC9710800; doi:10.1371/journal.pbio.3001897)
Supplement: S2 Table — A list of oligonucleotide pairs and primers used for deep sequencing. (DOCX) [file pbio.3001897.s011.docx]

| **Table S2. Primers used in this study** | | |
| --- | --- | --- |
| Name | Sequence | Description |
| Deep-F1 | ACACTCTTTCCCTACACGACGCTCTTCCGATCTNNNN gcgagaaaagccttgttt | Primers to amplify the random PAM for deep sequencing |
| Deep-R1 | ACTGGAGTTCAGACGTGTGCTCTTCCGATCTNNNN ctgaacttgtggccgtttac | Primers to amplify the random PAM for deep sequencing |
| P5-index1-F | AATGATACGGCGACCACCGAGATCTACAC TGAACCTT ACACTCTTTCCCTACACGAC | Primers to amplify the random PAM for deep sequencing |
| P7-adapter5-R | CAAGCAGAAGACGGCATACGAGAT GATCTG GTGACTGGAGTTCAGACGTGTG | Primers to amplify the random PAM for deep sequencing |
| P7-adapter6-R | CAAGCAGAAGACGGCATACGAGAT TACAAG GTGACTGGAGTTCAGACGTGTG | Primers to amplify the random PAM for deep sequencing |
| P7-adapter7-R | CAAGCAGAAGACGGCATACGAGAT CGTGAT GTGACTGGAGTTCAGACGTGTG | Primers to amplify the random PAM for deep sequencing |
| P7-adapter9-R | CAAGCAGAAGACGGCATACGAGAT TCAAGT GTGACTGGAGTTCAGACGTGTG | Primers to amplify the random PAM for deep sequencing |
| P7-adapter10-R | CAAGCAGAAGACGGCATACGAGAT CTGATC GTGACTGGAGTTCAGACGTGTG | Primers to amplify the random PAM for deep sequencing |
| P7-adapter11-R | CAAGCAGAAGACGGCATACGAGAT AAGCTA GTGACTGGAGTTCAGACGTGTG | Primers to amplify the random PAM for deep sequencing |
| P7-adapter12-R | CAAGCAGAAGACGGCATACGAGAT GTAGCC GTGACTGGAGTTCAGACGTGTG | Primers to amplify the random PAM for deep sequencing |
| P7-adapter13-R | CAAGCAGAAGACGGCATACGAGAT TTGACT GTGACTGGAGTTCAGACGTGTG | Primers to amplify the random PAM for deep sequencing |
| P7-adapter15-R | CAAGCAGAAGACGGCATACGAGAT TGACAT GTGACTGGAGTTCAGACGTGTG | Primers to amplify the random PAM for deep sequencing |
| P7-adapter16-R | CAAGCAGAAGACGGCATACGAGAT GGACGG GTGACTGGAGTTCAGACGTGTG | Primers to amplify the random PAM for deep sequencing |
| P7-adapter17-R | CAAGCAGAAGACGGCATACGAGAT CTCTAC GTGACTGGAGTTCAGACGTGTG | Primers to amplify the random PAM for deep sequencing |
| ANAPC15-sg1-F | cacc AGTGAGGGGAACAAAGTGGAC | Oligonucleotide pairs for construction of gRNA expression plasmid on Sa_tracr-gRNA vector |
| ANAPC15-sg1-R | aaac GTCCACTTTGTTCCCCTCACT | Oligonucleotide pairs for construction of gRNA expression plasmid on Sa_tracr-gRNA vector |
| ANAPC15-sg2-F | cacc GGGAAGAGTGAGGGGAACAAA | Oligonucleotide pairs for construction of gRNA expression plasmid on Sa_tracr-gRNA vector |
| ANAPC15-sg2-R | aaac TTTGTTCCCCTCACTCTTCCC | Oligonucleotide pairs for construction of gRNA expression plasmid on Sa_tracr-gRNA vector |
| EMX1-sg0-F | cacc ATAGGGTTAGGGGCCCCAGGC | Oligonucleotide pairs for construction of gRNA expression plasmid on Sa_tracr-gRNA vector |
| EMX1-sg0-R | aaac GCCTGGGGCCCCTAACCCTAT | Oligonucleotide pairs for construction of gRNA expression plasmid on Sa_tracr-gRNA vector |
| EMX1-sg1-F | cacc GGACCCAGGGGTAGAAATGGA | Oligonucleotide pairs for construction of gRNA expression plasmid on Sa_tracr-gRNA vector |
| EMX1-sg1-R | aaac TCCATTTCTACCCCTGGGTCC | Oligonucleotide pairs for construction of gRNA expression plasmid on Sa_tracr-gRNA vector |
| EMX1-sg2-F | cacc ACATTCACAGAAGGGGATGGC | Oligonucleotide pairs for construction of gRNA expression plasmid on Sa_tracr-gRNA vector |
| EMX1-sg2-R | aaac GCCATCCCCTTCTGTGAATGT | Oligonucleotide pairs for construction of gRNA expression plasmid on Sa_tracr-gRNA vector |
| EMX1-sg3-F | cacc GCCTGAGTCCGAGCAGAAGAAG | Oligonucleotide pairs for construction of gRNA expression plasmid on Sa_tracr-gRNA vector |
| EMX1-sg3-R | aaac CTTCTTCTGCTCGGACTCAGGC | Oligonucleotide pairs for construction of gRNA expression plasmid on Sa_tracr-gRNA vector |
| EMX1-sg4-F | cacc AAAGGTGAAAGAGAGATGGCT | Oligonucleotide pairs for construction of gRNA expression plasmid on Sa_tracr-gRNA vector |
| EMX1-sg4-R | aaac AGCCATCTCTCTTTCACCTTT | Oligonucleotide pairs for construction of gRNA expression plasmid on Sa_tracr-gRNA vector |
| EMX1-sg5-F | cacc GGAGATGGCACAGGAGAAGAT | Oligonucleotide pairs for construction of gRNA expression plasmid on Sa_tracr-gRNA vector |
| EMX1-sg5-R | aaac ATCTTCTCCTGTGCCATCTCC | Oligonucleotide pairs for construction of gRNA expression plasmid on Sa_tracr-gRNA vector |
| Emx1-sg6-F | cacc GCCTCCCGGCCCAGGTGAAGGT | Oligonucleotide pairs for construction of gRNA expression plasmid on Sa_tracr-gRNA vector |
| Emx1-sg6-R | aaac ACCTTCACCTGGGCCGGGAGGC | Oligonucleotide pairs for construction of gRNA expression plasmid on Sa_tracr-gRNA vector |
| EMX1-sg7-F | cacc GCAACTCTGCGGGGACTCCAGG | Oligonucleotide pairs for construction of gRNA expression plasmid on Sa_tracr-gRNA vector |
| EMX1-sg7-R | aaac CCTGGAGTCCCCGCAGAGTTGC | Oligonucleotide pairs for construction of gRNA expression plasmid on Sa_tracr-gRNA vector |
| GRIN2B-sg1-F | cacc AGATGCGGGTGATGATGCTCT | Oligonucleotide pairs for construction of gRNA expression plasmid on Sa_tracr-gRNA vector |
| GRIN2B-sg1-R | aaac AGAGCATCATCACCCGCATCT | Oligonucleotide pairs for construction of gRNA expression plasmid on Sa_tracr-gRNA vector |
| GRIN2B-sg2-F | cacc ACTTCCGACGAGGTGGCCATC | Oligonucleotide pairs for construction of gRNA expression plasmid on Sa_tracr-gRNA vector |
| GRIN2B-sg2-R | aaac GATGGCCACCTCGTCGGAAGT | Oligonucleotide pairs for construction of gRNA expression plasmid on Sa_tracr-gRNA vector |
| GRIN2B-sg3-F | cacc GCCACCATCTCTCCGTGGTACC | Oligonucleotide pairs for construction of gRNA expression plasmid on Sa_tracr-gRNA vector |
| GRIN2B-sg3-R | aaac GGTACCACGGAGAGATGGTGGC | Oligonucleotide pairs for construction of gRNA expression plasmid on Sa_tracr-gRNA vector |
| GRIN2B-sg4-F | cacc AGAGTAGGCTGGTAGATGGAG | Oligonucleotide pairs for construction of gRNA expression plasmid on Sa_tracr-gRNA vector |
| GRIN2B-sg4-R | aaac CTCCATCTACCAGCCTACTCT | Oligonucleotide pairs for construction of gRNA expression plasmid on Sa_tracr-gRNA vector |
| GRIN2B-sg5-F | cacc GTCAGACATGAGATCACAGAT | Oligonucleotide pairs for construction of gRNA expression plasmid on Sa_tracr-gRNA vector |
| GRIN2B-sg5-R | aaac ATCTGTGATCTCATGTCTGAC | Oligonucleotide pairs for construction of gRNA expression plasmid on Sa_tracr-gRNA vector |
| GRIN2B-sg6-F | cacc GTTCATGGCTACCAGTTCCACC | Oligonucleotide pairs for construction of gRNA expression plasmid on Sa_tracr-gRNA vector |
| GRIN2B-sg6-R | aaac GGTGGAACTGGTAGCCATGAAC | Oligonucleotide pairs for construction of gRNA expression plasmid on Sa_tracr-gRNA vector |
| GRIN2B-sg7-F | cacc AGGCTCAAAGGGAAGATACAT | Oligonucleotide pairs for construction of gRNA expression plasmid on Sa_tracr-gRNA vector |
| GRIN2B-sg7-R | aaac ATGTATCTTCCCTTTGAGCCT | Oligonucleotide pairs for construction of gRNA expression plasmid on Sa_tracr-gRNA vector |
| GRIN2B-sg8-F | cacc GATCTAACAAGGGAGAAAGTG | Oligonucleotide pairs for construction of gRNA expression plasmid on Sa_tracr-gRNA vector |
| GRIN2B-sg8-R | aaac CACTTTCTCCCTTGTTAGATC | Oligonucleotide pairs for construction of gRNA expression plasmid on Sa_tracr-gRNA vector |
| GRIN2B-sg9-F | cacc AAGGCTCATAGTAATCGTCTG | Oligonucleotide pairs for construction of gRNA expression plasmid on Sa_tracr-gRNA vector |
| GRIN2B-sg9-R | aaac CAGACGATTACTATGAGCCTT | Oligonucleotide pairs for construction of gRNA expression plasmid on Sa_tracr-gRNA vector |
| GRIN2B-sg10-F | cacc GGTGAGCCTCAGCTTCCCTGG | Oligonucleotide pairs for construction of gRNA expression plasmid on Sa_tracr-gRNA vector |
| GRIN2B-sg10-R | aaac CCAGGGAAGCTGAGGCTCACC | Oligonucleotide pairs for construction of gRNA expression plasmid on Sa_tracr-gRNA vector |
| GRIN2B-sg11-F | cacc GGGAAGCCTGCTGCAGCCACA | Oligonucleotide pairs for construction of gRNA expression plasmid on Sa_tracr-gRNA vector |
| GRIN2B-sg11-R | aaac TGTGGCTGCAGCAGGCTTCCC | Oligonucleotide pairs for construction of gRNA expression plasmid on Sa_tracr-gRNA vector |
| GRIN2B-sg17-F | cacc GTGTACTTAAAAGACATGCTT | Oligonucleotide pairs for construction of gRNA expression plasmid on Sa_tracr-gRNA vector |
| GRIN2B-sg17-R | aaac AAGCATGTCTTTTAAGTACAC | Oligonucleotide pairs for construction of gRNA expression plasmid on Sa_tracr-gRNA vector |
| Site3-sg-F | cacc AAGAATACTAAGCATAGACTC | Oligonucleotide pairs for construction of gRNA expression plasmid on Sa_tracr-gRNA vector |
| Site3-sg-R | aaac GAGTCTATGCTTAGTATTCTT | Oligonucleotide pairs for construction of gRNA expression plasmid on Sa_tracr-gRNA vector |
| RUNX1-sg13-F | cacc GAAAGAGAGATGTAGGGCTAG | Oligonucleotide pairs for construction of gRNA expression plasmid on Sa_tracr-gRNA vector |
| RUNX1-sg13-R | aaac CTAGCCCTACATCTCTCTTTC | Oligonucleotide pairs for construction of gRNA expression plasmid on Sa_tracr-gRNA vector |
| T7-ANAPC15-sg1/2-F | TGTGTGGAGGAGGTAGGGATAG | Primers to amplify the Endogenous target site for T7E1 |
| T7-ANAPC15-sg1/2-R | GCTCCCAGCCCGATTATTCC | Primers to amplify the Endogenous target site for T7E1 |
| T7-EMX1-sg41/6/7-F | TCCCCACGGATTCCATCATTC | Primers to amplify the Endogenous target site for T7E1 |
| T7-EMX1-sg41/6/7-R | CTCTGCTCCGGCCAGTTTTT | Primers to amplify the Endogenous target site for T7E1 |
| T7-EMX1-F0 | ACGCAGTGGGTCATAGGCTC | Primers to amplify the Endogenous target site for T7E1 |
| T7-EMX1-R0 | GGACTCAGGCCCTTCCTCCT | Primers to amplify the Endogenous target site for T7E1 |
| T7-EMX1-F1 | CTTCGTGAGTGGCTTCCCTG | Primers to amplify the Endogenous target site for T7E1 |
| T7-EMX1-R1 | CTTTCCTGGGAGGGAGACCT | Primers to amplify the Endogenous target site for T7E1 |
| T7-EMX1-primer-F4 | GATGGGCATCCAAGTCTGGT | Primers to amplify the Endogenous target site for T7E1 |
| T7-EMX1-primer-R4 | TAGCATTCACCTGCGATGGG | Primers to amplify the Endogenous target site for T7E1 |
| T7-EMX1-primer-F5 | AGGCTAGGCTGTATCAGCC | Primers to amplify the Endogenous target site for T7E1 |
| T7-EMX1-primer-R5 | AACAGCGGAGGATCAGTAACT | Primers to amplify the Endogenous target site for T7E1 |
| T7-GRIN2B-primer-F1/2/3/4/5/6 | CAGGGAGTCGACGAGTTGAA | Primers to amplify the Endogenous target site for T7E1 |
| T7-GRIN2B-primer-R1/2/3/4/5/6 | TAATTGCTGGCCTATCCACGC | Primers to amplify the Endogenous target site for T7E1 |
| T7-GRIN2B-primer-F7 | ACTTCACTCACTGCCTGTCA | Primers to amplify the Endogenous target site for T7E1 |
| T7-GRIN2B-primer-R7 | GCACAAAGTGAGTGTGTGGG | Primers to amplify the Endogenous target site for T7E1 |
| T7-GRIN2B-primer-F8 | GGGGCTTTGAGAGTGTTCCC | Primers to amplify the Endogenous target site for T7E1 |
| T7-GRIN2B-primer-R8 | AAGTCACTGCCCACCTACCT | Primers to amplify the Endogenous target site for T7E1 |
| T7-GRIN2B-primer-F9 | AGAGAGTGCCAGCAGCAAAC | Primers to amplify the Endogenous target site for T7E1 |
| T7-GRIN2B-primer-R9 | GTGCTGAACTGCTGAACTGTG | Primers to amplify the Endogenous target site for T7E1 |
| T7-GRIN2B-primer-F10/11 | GCCTAACATTCAGCAGGTGAA | Primers to amplify the Endogenous target site for T7E1 |
| T7-GRIN2B-primer-R10/11 | ATCCAGGATCTCAGGGAGAAT | Primers to amplify the Endogenous target site for T7E1 |
| T7-Site3-F | GGGAAGTCCTTCCATTAGGT | Primers to amplify the Endogenous target site for T7E1 |
| T7-Site3-R | TGTAGTATTTAAGCCCTGGGT | Primers to amplify the Endogenous target site for T7E1 |
| EMX1-deep-F0 | ACACTCTTTCCCTACACGACGCTCTTCCGATCTNNNNgaggaacaggaaaaccaccct | Primers to amplify the Endogenous target site for deep sequencing of step I |
| EMX1-deep-R0 | ACTGGAGTTCAGACGTGTGCTCTTCCGATCTNNNNcaccttcacctgggccag | Primers to amplify the Endogenous target site for deep sequencing of step I |
| EMX1-deep-F4 | ACACTCTTTCCCTACACGACGCTCTTCCGATCTNNNNgggcaagtgagagaattggag | Primers to amplify the Endogenous target site for deep sequencing of step I |
| EMX1-deep-R4 | ACTGGAGTTCAGACGTGTGCTCTTCCGATCTNNNNacaagccaggcaatgtttct | Primers to amplify the Endogenous target site for deep sequencing of step I |
| EMX1-deep-F7 | ACACTCTTTCCCTACACGACGCTCTTCCGATCTNNNNtcggagcagtcgagtggaaa | Primers to amplify the Endogenous target site for deep sequencing of step I |
| EMX1-deep-R7 | ACTGGAGTTCAGACGTGTGCTCTTCCGATCTNNNNcagagagttccgcttcctcg | Primers to amplify the Endogenous target site for deep sequencing of step I |
| GRIN2B-deep-F1/2/3/5/6 | ACACTCTTTCCCTACACGACGCTCTTCCGATCTNNNNtgtcaggcagcagagctc | Primers to amplify the Endogenous target site for deep sequencing of step I |
| GRIN2B-deep-R1/2/3/5/6 | ACTGGAGTTCAGACGTGTGCTCTTCCGATCTNNNNggcgatggcttcctggtc | Primers to amplify the Endogenous target site for deep sequencing of step I |
| GRIN2B-deep-F4/26 | ACACTCTTTCCCTACACGACGCTCTTCCGATCTNNNNtgcctgtagctgccaatgac | Primers to amplify the Endogenous target site for deep sequencing of step I |
| GRIN2B-deep-R4/26 | ACTGGAGTTCAGACGTGTGCTCTTCCGATCTNNNNcttcaactcgtcgactccct | Primers to amplify the Endogenous target site for deep sequencing of step I |
| GRIN2B-deep-F7 | ACACTCTTTCCCTACACGACGCTCTTCCGATCTNNNNattaagggatggtgcttggaa | Primers to amplify the Endogenous target site for deep sequencing of step I |
| GRIN2B-deep-R7 | ACTGGAGTTCAGACGTGTGCTCTTCCGATCTNNNNatgctggtaactggtacaggg | Primers to amplify the Endogenous target site for deep sequencing of step I |
| GRIN2B-deep-F8 | ACACTCTTTCCCTACACGACGCTCTTCCGATCTNNNNcagctcttaagtggaagcca | Primers to amplify the Endogenous target site for deep sequencing of step I |
| GRIN2B-deep-R8 | ACTGGAGTTCAGACGTGTGCTCTTCCGATCTNNNNgcgctccaccatttcatac | Primers to amplify the Endogenous target site for deep sequencing of step I |
| GRIN2B-deep-F9 | ACACTCTTTCCCTACACGACGCTCTTCCGATCTNNNNtcaacaacaggggaataggaca | Primers to amplify the Endogenous target site for deep sequencing of step I |
| GRIN2B-deep-R9 | ACTGGAGTTCAGACGTGTGCTCTTCCGATCTNNNNtgcaatcatccacaagagca | Primers to amplify the Endogenous target site for deep sequencing of step I |
| GRIN2B-deep-F10/11 | ACACTCTTTCCCTACACGACGCTCTTCCGATCTNNNNgaccacccaagaagggcata | Primers to amplify the Endogenous target site for deep sequencing of step I |
| GRIN2B-deep-R10/11 | ACTGGAGTTCAGACGTGTGCTCTTCCGATCTNNNNaagcacctagcagttctttcca | Primers to amplify the Endogenous target site for deep sequencing of step I |
| Site7-deep-F | ACACTCTTTCCCTACACGACGCTCTTCCGATCTNNNNGATGCCCTCCATCTTCTCCG | Primers to amplify the Endogenous target site for deep sequencing of step I |
| Site7-deep-R | ACTGGAGTTCAGACGTGTGCTCTTCCGATCT AGGTTTGCATAGACCTGCCC | Primers to amplify the Endogenous target site for deep sequencing of step I |
| P5-index1-F | AATGATACGGCGACCACCGAGATCTACAC TGAACCTT ACACTCTTTCCCTACACGAC | Primers to amplify the Endogenous target site for deep sequencing of step II |
| P5-index2-F | AATGATACGGCGACCACCGAGATCTACAC TGCTAAGT ACACTCTTTCCCTACACGAC | Primers to amplify the Endogenous target site for deep sequencing of step II |
| P5-index3-F | AATGATACGGCGACCACCGAGATCTACAC TAAGACAC ACACTCTTTCCCTACACGAC | Primers to amplify the Endogenous target site for deep sequencing of step II |
| P5-index5-F | AATGATACGGCGACCACCGAGATCTACAC CTAATCGA ACACTCTTTCCCTACACGAC | Primers to amplify the Endogenous target site for deep sequencing of step II |
| P5-index6-F | AATGATACGGCGACCACCGAGATCTACAC CTAGAACA ACACTCTTTCCCTACACGAC | Primers to amplify the Endogenous target site for deep sequencing of step II |
| P5-index7-F | AATGATACGGCGACCACCGAGATCTACAC TAAGTTCC ACACTCTTTCCCTACACGAC | Primers to amplify the Endogenous target site for deep sequencing of step II |
| P5-index8-F | AATGATACGGCGACCACCGAGATCTACAC TAGACCTA ACACTCTTTCCCTACACGAC | Primers to amplify the Endogenous target site for deep sequencing of step II |
| P5-index9-F | AATGATACGGCGACCACCGAGATCTACAC CGATGT ACACTCTTTCCCTACACGAC | Primers to amplify the Endogenous target site for deep sequencing of step II |
| P5-index10-F | AATGATACGGCGACCACCGAGATCTACAC TGACCA ACACTCTTTCCCTACACGAC | Primers to amplify the Endogenous target site for deep sequencing of step II |
| P5-index15-F | AATGATACGGCGACCACCGAGATCTACAC ATCACG ACACTCTTTCCCTACACGAC | Primers to amplify the Endogenous target site for deep sequencing of step II |
| P5-index16-F | AATGATACGGCGACCACCGAGATCTACAC TTAGGC ACACTCTTTCCCTACACGAC | Primers to amplify the Endogenous target site for deep sequencing of step II |
| P5-index17-F | AATGATACGGCGACCACCGAGATCTACAC ACTTGA ACACTCTTTCCCTACACGAC | Primers to amplify the Endogenous target site for deep sequencing of step II |
| P5-index18-F | AATGATACGGCGACCACCGAGATCTACAC GATCAG ACACTCTTTCCCTACACGAC | Primers to amplify the Endogenous target site for deep sequencing of step II |
| P5-index19-F | AATGATACGGCGACCACCGAGATCTACAC TAGCTT ACACTCTTTCCCTACACGAC | Primers to amplify the Endogenous target site for deep sequencing of step II |
| P5-index20-F | AATGATACGGCGACCACCGAGATCTACAC GGCTAC ACACTCTTTCCCTACACGAC | Primers to amplify the Endogenous target site for deep sequencing of step II |
| P5-index21-F | AATGATACGGCGACCACCGAGATCTACAC AGTCAA ACACTCTTTCCCTACACGAC | Primers to amplify the Endogenous target site for deep sequencing of step II |
| P5-index22-F | AATGATACGGCGACCACCGAGATCTACAC AGTTCC ACACTCTTTCCCTACACGAC | Primers to amplify the Endogenous target site for deep sequencing of step II |
| P5-index23-F | AATGATACGGCGACCACCGAGATCTACAC ATGTCA ACACTCTTTCCCTACACGAC | Primers to amplify the Endogenous target site for deep sequencing of step II |
| P5-index24-F | AATGATACGGCGACCACCGAGATCTACAC CCGTCC ACACTCTTTCCCTACACGAC | Primers to amplify the Endogenous target site for deep sequencing of step II |
| P5-index25-F | AATGATACGGCGACCACCGAGATCTACAC GTAGAG ACACTCTTTCCCTACACGAC | Primers to amplify the Endogenous target site for deep sequencing of step II |
| P5-index26-F | AATGATACGGCGACCACCGAGATCTACAC GTCCGC ACACTCTTTCCCTACACGAC | Primers to amplify the Endogenous target site for deep sequencing of step II |
| P5-index27-F | AATGATACGGCGACCACCGAGATCTACAC GTGAAA ACACTCTTTCCCTACACGAC | Primers to amplify the Endogenous target site for deep sequencing of step II |
| P5-index28-F | AATGATACGGCGACCACCGAGATCTACACGTGGCCACACTCTTTCCCTACACGAC | Primers to amplify the Endogenous target site for deep sequencing of step II |
| P5-index29-F | AATGATACGGCGACCACCGAGATCTACACGTTTCGACACTCTTTCCCTACACGAC | Primers to amplify the Endogenous target site for deep sequencing of step II |
| P5-index30-F | AATGATACGGCGACCACCGAGATCTACACCGTACGACACTCTTTCCCTACACGAC | Primers to amplify the Endogenous target site for deep sequencing of step II |
| P5-index31-F | AATGATACGGCGACCACCGAGATCTACACGAGTGGACACTCTTTCCCTACACGAC | Primers to amplify the Endogenous target site for deep sequencing of step II |
| P5-index32-F | AATGATACGGCGACCACCGAGATCTACACGGTAGCACACTCTTTCCCTACACGAC | Primers to amplify the Endogenous target site for deep sequencing of step II |
| P5-index33-F | AATGATACGGCGACCACCGAGATCTACACACTGATACACTCTTTCCCTACACGAC | Primers to amplify the Endogenous target site for deep sequencing of step II |
| P5-index34-F | AATGATACGGCGACCACCGAGATCTACACATGAGCACACTCTTTCCCTACACGAC | Primers to amplify the Endogenous target site for deep sequencing of step II |
| P5-index35-F | AATGATACGGCGACCACCGAGATCTACACATTCCTACACTCTTTCCCTACACGAC | Primers to amplify the Endogenous target site for deep sequencing of step II |
| P5-index36-F | AATGATACGGCGACCACCGAGATCTACACCAAAAGACACTCTTTCCCTACACGAC | Primers to amplify the Endogenous target site for deep sequencing of step II |
| P5-index37-F | AATGATACGGCGACCACCGAGATCTACAC TATAGCCT ACACTCTTTCCCTACACGAC | Primers to amplify the Endogenous target site for deep sequencing of step II |
| P5-index38-F | AATGATACGGCGACCACCGAGATCTACAC ATAGAGGC ACACTCTTTCCCTACACGAC | Primers to amplify the Endogenous target site for deep sequencing of step II |
| P5-index39-F | AATGATACGGCGACCACCGAGATCTACAC CCTATCCT ACACTCTTTCCCTACACGAC | Primers to amplify the Endogenous target site for deep sequencing of step II |
| P5-index40-F | AATGATACGGCGACCACCGAGATCTACAC GGCTCTGA ACACTCTTTCCCTACACGAC | Primers to amplify the Endogenous target site for deep sequencing of step II |
| P5-index41-F | AATGATACGGCGACCACCGAGATCTACAC AGGCGAAG ACACTCTTTCCCTACACGAC | Primers to amplify the Endogenous target site for deep sequencing of step II |
| P5-index42-F | AATGATACGGCGACCACCGAGATCTACAC TAATCTTA ACACTCTTTCCCTACACGAC | Primers to amplify the Endogenous target site for deep sequencing of step II |
| P5-index43-F | AATGATACGGCGACCACCGAGATCTACAC CAGGACGT ACACTCTTTCCCTACACGAC | Primers to amplify the Endogenous target site for deep sequencing of step II |
| P5-index44-F | AATGATACGGCGACCACCGAGATCTACAC GTACTGAC ACACTCTTTCCCTACACGAC | Primers to amplify the Endogenous target site for deep sequencing of step II |
| P7-adapter3-R | CAAGCAGAAGACGGCATACGAGAT CACTGT GTGACTGGAGTTCAGACGTGTG | Primers to amplify the Endogenous target site for deep sequencing of step II |
| P7-adapter4-R | CAAGCAGAAGACGGCATACGAGAT ATTGGC GTGACTGGAGTTCAGACGTGTG | Primers to amplify the Endogenous target site for deep sequencing of step II |
| P7-adapter5-R | CAAGCAGAAGACGGCATACGAGAT GATCTG GTGACTGGAGTTCAGACGTGTG | Primers to amplify the Endogenous target site for deep sequencing of step II |
| P7-adapter6-R | CAAGCAGAAGACGGCATACGAGAT TACAAG GTGACTGGAGTTCAGACGTGTG | Primers to amplify the Endogenous target site for deep sequencing of step II |
| P7-adapter8-R | CAAGCAGAAGACGGCATACGAGAT GCCTAA GTGACTGGAGTTCAGACGTGTG | Primers to amplify the Endogenous target site for deep sequencing of step II |
| P7-adapter9-R | CAAGCAGAAGACGGCATACGAGAT TCAAGT GTGACTGGAGTTCAGACGTGTG | Primers to amplify the Endogenous target site for deep sequencing of step II |
| P7-adapter10-R | CAAGCAGAAGACGGCATACGAGAT CTGATC GTGACTGGAGTTCAGACGTGTG | Primers to amplify the Endogenous target site for deep sequencing of step II |
| P7-adapter11-R | CAAGCAGAAGACGGCATACGAGAT AAGCTA GTGACTGGAGTTCAGACGTGTG | Primers to amplify the Endogenous target site for deep sequencing of step II |
| P7-adapter12-R | CAAGCAGAAGACGGCATACGAGAT GTAGCC GTGACTGGAGTTCAGACGTGTG | Primers to amplify the Endogenous target site for deep sequencing of step II |
| P7-adapter13-R | CAAGCAGAAGACGGCATACGAGAT TTGACT GTGACTGGAGTTCAGACGTGTG | Primers to amplify the Endogenous target site for deep sequencing of step II |
| P7-adapter14-R | CAAGCAGAAGACGGCATACGAGAT GGAACT GTGACTGGAGTTCAGACGTGTG | Primers to amplify the Endogenous target site for deep sequencing of step II |
| P7-adapter15-R | CAAGCAGAAGACGGCATACGAGAT TGACAT GTGACTGGAGTTCAGACGTGTG | Primers to amplify the Endogenous target site for deep sequencing of step II |
| P7-adapter16-R | CAAGCAGAAGACGGCATACGAGAT GGACGG GTGACTGGAGTTCAGACGTGTG | Primers to amplify the Endogenous target site for deep sequencing of step II |
| P7-adapter17-R | CAAGCAGAAGACGGCATACGAGAT CTCTAC GTGACTGGAGTTCAGACGTGTG | Primers to amplify the Endogenous target site for deep sequencing of step II |
| P7-adapter18-R | CAAGCAGAAGACGGCATACGAGAT GCGGAC GTGACTGGAGTTCAGACGTGTG | Primers to amplify the Endogenous target site for deep sequencing of step II |
| P7-adapter19-R | CAAGCAGAAGACGGCATACGAGAT TTTCAC GTGACTGGAGTTCAGACGTGTG | Primers to amplify the Endogenous target site for deep sequencing of step II |
| P7-adapter20-R | CAAGCAGAAGACGGCATACGAGAT GGCCAC GTGACTGGAGTTCAGACGTGTG | Primers to amplify the Endogenous target site for deep sequencing of step II |
| P7-adapter21-R | CAAGCAGAAGACGGCATACGAGAT CGAAAC GTGACTGGAGTTCAGACGTGTG | Primers to amplify the Endogenous target site for deep sequencing of step II |
| P7-adapter22-R | CAAGCAGAAGACGGCATACGAGAT CGTACG GTGACTGGAGTTCAGACGTGTG | Primers to amplify the Endogenous target site for deep sequencing of step II |
| P7-adapter23-R | CAAGCAGAAGACGGCATACGAGAT CCACTC GTGACTGGAGTTCAGACGTGTG | Primers to amplify the Endogenous target site for deep sequencing of step II |
| P7-adapter24-R | CAAGCAGAAGACGGCATACGAGAT GCTACC GTGACTGGAGTTCAGACGTGTG | Primers to amplify the Endogenous target site for deep sequencing of step II |
| P7-adapter25-R | CAAGCAGAAGACGGCATACGAGAT CGAGTAAT GTGACTGGAGTTCAGACGTGTG | Primers to amplify the Endogenous target site for deep sequencing of step II |
| P7-adapter26-R | CAAGCAGAAGACGGCATACGAGAT TCTCCGGA GTGACTGGAGTTCAGACGTGTG | Primers to amplify the Endogenous target site for deep sequencing of step II |
| P7-adapter27-R | CAAGCAGAAGACGGCATACGAGAT AATGAGCG GTGACTGGAGTTCAGACGTGTG | Primers to amplify the Endogenous target site for deep sequencing of step II |
| P7-adapter28-R | CAAGCAGAAGACGGCATACGAGAT GGAATCTC GTGACTGGAGTTCAGACGTGTG | Primers to amplify the Endogenous target site for deep sequencing of step II |
| P7-adapter29-R | CAAGCAGAAGACGGCATACGAGAT TTCTGAAT GTGACTGGAGTTCAGACGTGTG | Primers to amplify the Endogenous target site for deep sequencing of step II |
| P7-adapter30-R | CAAGCAGAAGACGGCATACGAGAT ACGAATTC GTGACTGGAGTTCAGACGTGTG | Primers to amplify the Endogenous target site for deep sequencing of step II |
| P7-adapter31-R | CAAGCAGAAGACGGCATACGAGAT AGCTTCAG GTGACTGGAGTTCAGACGTGTG | Primers to amplify the Endogenous target site for deep sequencing of step II |
| P7-adapter32-R | CAAGCAGAAGACGGCATACGAGAT GCGCATTA GTGACTGGAGTTCAGACGTGTG | Primers to amplify the Endogenous target site for deep sequencing of step II |
| P7-adapter33-R | CAAGCAGAAGACGGCATACGAGAT CATAGCCG GTGACTGGAGTTCAGACGTGTG | Primers to amplify the Endogenous target site for deep sequencing of step II |
| P7-adapter34-R | CAAGCAGAAGACGGCATACGAGAT TTCGCGGA GTGACTGGAGTTCAGACGTGTG | Primers to amplify the Endogenous target site for deep sequencing of step II |
| P7-adapter35-R | CAAGCAGAAGACGGCATACGAGAT GCGCGAGA GTGACTGGAGTTCAGACGTGTG | Primers to amplify the Endogenous target site for deep sequencing of step II |
| P7-adapter36-R | CAAGCAGAAGACGGCATACGAGAT CTATCGCT GTGACTGGAGTTCAGACGTGTG | Primers to amplify the Endogenous target site for deep sequencing of step II |
| P7-adapter37-R | CAAGCAGAAGACGGCATACGAGAT GTCGTGAT GTGACTGGAGTTCAGACGTGTG | Primers to amplify the Endogenous target site for deep sequencing of step II |
| P7-adapter38-R | CAAGCAGAAGACGGCATACGAGAT ACCACTGT GTGACTGGAGTTCAGACGTGTG | Primers to amplify the Endogenous target site for deep sequencing of step II |
| P7-adapter39-R | CAAGCAGAAGACGGCATACGAGAT TGGATCTG GTGACTGGAGTTCAGACGTGTG | Primers to amplify the Endogenous target site for deep sequencing of step II |
| P7-adapter40-R | CAAGCAGAAGACGGCATACGAGAT CCGTTTGT GTGACTGGAGTTCAGACGTGTG | Primers to amplify the Endogenous target site for deep sequencing of step II |
| P7-adapter41-R | CAAGCAGAAGACGGCATACGAGAT TGCTGGGT GTGACTGGAGTTCAGACGTGTG | Primers to amplify the Endogenous target site for deep sequencing of step II |
| P7-adapter42-R | CAAGCAGAAGACGGCATACGAGAT GAGGGGTT GTGACTGGAGTTCAGACGTGTG | Primers to amplify the Endogenous target site for deep sequencing of step II |
| P7-adapter43-R | CAAGCAGAAGACGGCATACGAGAT AGGTTGGG GTGACTGGAGTTCAGACGTGTG | Primers to amplify the Endogenous target site for deep sequencing of step II |
| P7-adapter44-R | CAAGCAGAAGACGGCATACGAGAT GTGTGGTG GTGACTGGAGTTCAGACGTGTG | Primers to amplify the Endogenous target site for deep sequencing of step II |
| P7-adapter45-R | CAAGCAGAAGACGGCATACGAGAT TGGGTTTC GTGACTGGAGTTCAGACGTGTG | Primers to amplify the Endogenous target site for deep sequencing of step II |
| P7-adapter46-R | CAAGCAGAAGACGGCATACGAGAT TGGTCACA GTGACTGGAGTTCAGACGTGTG | Primers to amplify the Endogenous target site for deep sequencing of step II |
| P7-adapter47-R | CAAGCAGAAGACGGCATACGAGAT TTGACCCT GTGACTGGAGTTCAGACGTGTG | Primers to amplify the Endogenous target site for deep sequencing of step II |
| P7-adapter48-R | CAAGCAGAAGACGGCATACGAGAT CCACTCCT GTGACTGGAGTTCAGACGTGTG | Primers to amplify the Endogenous target site for deep sequencing of step II |
|  |  |  |
| P5-index1-F | AATGATACGGCGACCACCGAGATCTACAC TGAACCTT ACACTCTTTCCCTACACGAC | Primers to amplify the GUIDE-seq site for deep sequencing of step I |
| P5-index2-F | AATGATACGGCGACCACCGAGATCTACAC TGCTAAGT ACACTCTTTCCCTACACGAC | Primers to amplify the GUIDE-seq site for deep sequencing of step I |
| P5-index3-F | AATGATACGGCGACCACCGAGATCTACAC TAAGACAC ACACTCTTTCCCTACACGAC | Primers to amplify the GUIDE-seq site for deep sequencing of step I |
| Nuclease_off_-_GSP1 | GGATCTCGACGCTCTCCCTGTTTAATTGAGTTGTCATATGTTAATAAC | Primers to amplify the GUIDE-seq site for deep sequencing of step I |
| P5_2 | AATGATACGGCGACCACCGAGATCTACAC | Primers to amplify the GUIDE-seq site for deep sequencing of step II |
| Nuclease_off_-_GSP2 | CAAGCAGAAGACGGCATACGAGATCTAGTACGGTGACTGGAGTTCAGACGTGTGCTCTTCCGATCTGAGTTGTCATATGTTAATAACGG | Primers to amplify the GUIDE-seq site for deep sequencing of step II |
|  |  |  |
| ITIH5-F | cacc GGCTCGGAGATCATCATTGCG | Oligonucleotide pairs for construction of off-target site expression plasmid on Sa_tracr-gRNA vector |
| ITIH5-R | aaac CGCAATGATGATCTCCGAGCC | Oligonucleotide pairs for construction of off-target site expression plasmid on Sa_tracr-gRNA vector |
| ITIH5-OT1-2-F | cacc AACTCGGAGATCATCATTGCG | Oligonucleotide pairs for construction of off-target site expression plasmid on Sa_tracr-gRNA vector |
| ITIH5-OT2-3-F | cacc GATTCGGAGATCATCATTGCG | Oligonucleotide pairs for construction of off-target site expression plasmid on Sa_tracr-gRNA vector |
| ITIH5-OT3-4-F | cacc GGTCCGGAGATCATCATTGCG | Oligonucleotide pairs for construction of off-target site expression plasmid on Sa_tracr-gRNA vector |
| ITIH5-OT4-5-F | cacc GGCCTGGAGATCATCATTGCG | Oligonucleotide pairs for construction of off-target site expression plasmid on Sa_tracr-gRNA vector |
| ITIH5-OT5-6-F | cacc GGCTTAGAGATCATCATTGCG | Oligonucleotide pairs for construction of off-target site expression plasmid on Sa_tracr-gRNA vector |
| ITIH5-OT6-7-F | cacc GGCTCAAAGATCATCATTGCG | Oligonucleotide pairs for construction of off-target site expression plasmid on Sa_tracr-gRNA vector |
| ITIH5-OT7-8-F | cacc GGCTCGAGGATCATCATTGCG | Oligonucleotide pairs for construction of off-target site expression plasmid on Sa_tracr-gRNA vector |
| ITIH5-OT8-9-F | cacc GGCTCGGGAATCATCATTGCG | Oligonucleotide pairs for construction of off-target site expression plasmid on Sa_tracr-gRNA vector |
| ITIH5-OT9-10-F | cacc GGCTCGGAAGTCATCATTGCG | Oligonucleotide pairs for construction of off-target site expression plasmid on Sa_tracr-gRNA vector |
| ITIH5-OT10-11-F | cacc GGCTCGGAGGCCATCATTGCG | Oligonucleotide pairs for construction of off-target site expression plasmid on Sa_tracr-gRNA vector |
| ITIH5-OT11-12-F | cacc GGCTCGGAGACTATCATTGCG | Oligonucleotide pairs for construction of off-target site expression plasmid on Sa_tracr-gRNA vector |
| ITIH5-OT12-13-F | cacc GGCTCGGAGATTGTCATTGCG | Oligonucleotide pairs for construction of off-target site expression plasmid on Sa_tracr-gRNA vector |
| ITIH5-OT13-14-F | cacc GGCTCGGAGATCGCCATTGCG | Oligonucleotide pairs for construction of off-target site expression plasmid on Sa_tracr-gRNA vector |
| ITIH5-OT14-15-F | cacc GGCTCGGAGATCACTATTGCG | Oligonucleotide pairs for construction of off-target site expression plasmid on Sa_tracr-gRNA vector |
| ITIH5-OT15-16-F | cacc GGCTCGGAGATCATTGTTGCG | Oligonucleotide pairs for construction of off-target site expression plasmid on Sa_tracr-gRNA vector |
| ITIH5-OT16-17-F | cacc GGCTCGGAGATCATCGCTGCG | Oligonucleotide pairs for construction of off-target site expression plasmid on Sa_tracr-gRNA vector |
| ITIH5-OT17-18-F | cacc GGCTCGGAGATCATCACCGCG | Oligonucleotide pairs for construction of off-target site expression plasmid on Sa_tracr-gRNA vector |
| ITIH5-OT18-19-F | cacc GGCTCGGAGATCATCATCACG | Oligonucleotide pairs for construction of off-target site expression plasmid on Sa_tracr-gRNA vector |
| ITIH5-OT19-20-F | cacc GGCTCGGAGATCATCATTATG | Oligonucleotide pairs for construction of off-target site expression plasmid on Sa_tracr-gRNA vector |
| ITIH5-OT20-21-F | cacc GGCTCGGAGATCATCATTGTA | Oligonucleotide pairs for construction of off-target site expression plasmid on Sa_tracr-gRNA vector |
| ITIH5-OT1-2-R | aaac CGCAATGATGATCTCCGAGTT | Oligonucleotide pairs for construction of off-target site expression plasmid on Sa_tracr-gRNA vector |
| ITIH5-OT2-3-R | aaac CGCAATGATGATCTCCGAATC | Oligonucleotide pairs for construction of off-target site expression plasmid on Sa_tracr-gRNA vector |
| ITIH5-OT3-4-R | aaac CGCAATGATGATCTCCGGACC | Oligonucleotide pairs for construction of off-target site expression plasmid on Sa_tracr-gRNA vector |
| ITIH5-OT4-5-R | aaac CGCAATGATGATCTCCAGGCC | Oligonucleotide pairs for construction of off-target site expression plasmid on Sa_tracr-gRNA vector |
| ITIH5-OT5-6-R | aaac CGCAATGATGATCTCTAAGCC | Oligonucleotide pairs for construction of off-target site expression plasmid on Sa_tracr-gRNA vector |
| ITIH5-OT6-7-R | aaac CGCAATGATGATCTTTGAGCC | Oligonucleotide pairs for construction of off-target site expression plasmid on Sa_tracr-gRNA vector |
| ITIH5-OT7-8-R | aaac CGCAATGATGATCCTCGAGCC | Oligonucleotide pairs for construction of off-target site expression plasmid on Sa_tracr-gRNA vector |
| ITIH5-OT8-9-R | aaac CGCAATGATGATTCCCGAGCC | Oligonucleotide pairs for construction of off-target site expression plasmid on Sa_tracr-gRNA vector |
| ITIH5-OT9-10-R | aaac CGCAATGATGACTTCCGAGCC | Oligonucleotide pairs for construction of off-target site expression plasmid on Sa_tracr-gRNA vector |
| ITIH5-OT10-11-R | aaac CGCAATGATGGCCTCCGAGCC | Oligonucleotide pairs for construction of off-target site expression plasmid on Sa_tracr-gRNA vector |
| ITIH5-OT11-12-R | aaac CGCAATGATAGTCTCCGAGCC | Oligonucleotide pairs for construction of off-target site expression plasmid on Sa_tracr-gRNA vector |
| ITIH5-OT12-13-R | aaac CGCAATGACAATCTCCGAGCC | Oligonucleotide pairs for construction of off-target site expression plasmid on Sa_tracr-gRNA vector |
| ITIH5-OT13-14-R | aaac CGCAATGGCGATCTCCGAGCC | Oligonucleotide pairs for construction of off-target site expression plasmid on Sa_tracr-gRNA vector |
| ITIH5-OT14-15-R | aaac CGCAATAGTGATCTCCGAGCC | Oligonucleotide pairs for construction of off-target site expression plasmid on Sa_tracr-gRNA vector |
| ITIH5-OT15-16-R | aaac CGCAACAATGATCTCCGAGCC | Oligonucleotide pairs for construction of off-target site expression plasmid on Sa_tracr-gRNA vector |
| ITIH5-OT16-17-R | aaac CGCAGCGATGATCTCCGAGCC | Oligonucleotide pairs for construction of off-target site expression plasmid on Sa_tracr-gRNA vector |
| ITIH5-OT17-18-R | aaac CGCGGTGATGATCTCCGAGCC | Oligonucleotide pairs for construction of off-target site expression plasmid on Sa_tracr-gRNA vector |
| ITIH5-OT18-19-R | aaac CGTGATGATGATCTCCGAGCC | Oligonucleotide pairs for construction of off-target site expression plasmid on Sa_tracr-gRNA vector |
| ITIH5-OT19-20-R | aaac CATAATGATGATCTCCGAGCC | Oligonucleotide pairs for construction of off-target site expression plasmid on Sa_tracr-gRNA vector |
| ITIH5-OT20-21-R | aaac TACAATGATGATCTCCGAGCC | Oligonucleotide pairs for construction of off-target site expression plasmid on Sa_tracr-gRNA vector |
