# Supplementary material for: Identification of SaCas9 orthologs containing a conserved serine residue that determines simple NNGG PAM recognition
Source: PLoS Biol. 2022 Nov 30;20(11):e3001897. doi: 10.1371/journal.pbio.3001897 (PMC9710800; doi:10.1371/journal.pbio.3001897)
Supplement: S3 Table — A list of the endogenous target sites of human and their downstream PAM. PAM, protospacer adjacent motif. (DOCX) [file pbio.3001897.s012.docx]

| Table S3. Target sites used in this study | | | | |
| --- | --- | --- | --- | --- |
| Gene | Name | Targets | PAM | Description |
| ANAPC15 | A1 | AGTGAGGGGAACAAAGTGGAC | ATGG | Endogenous target site of human |
| ANAPC15 | A2 | GGGAAGAGTGAGGGGAACAAA | GTGG | Endogenous target site of human |
| EMX1 | E0(EMX1_1) | ATAGGGTTAGGGGCCCCAGGC | CGGG | Endogenous target site of human |
| EMX1 | E1 | GGACCCAGGGGTAGAAATGGA | GAGG | Endogenous target site of human |
| EMX1 | E2(EMX1_2) | ACATTCACAGAAGGGGATGGC | AGGG | Endogenous target site of human |
| EMX1 | E4 | AAAGGTGAAAGAGAGATGGCT | GGGG | Endogenous target site of human |
| EMX1 | E5 | GGAGATGGCACAGGAGAAGAT | TGGG | Endogenous target site of human |
| EMX1 | E7 | CAACTCTGCGGGGACTCCAGG | CCGG | Endogenous target site of human |
| GRIN2B | G1 | AGATGCGGGTGATGATGCTCT | TTGG | Endogenous target site of human |
| GRIN2B | G2 | ACTTCCGACGAGGTGGCCATC | AAGG | Endogenous target site of human |
| GRIN2B | G3 | CCACCATCTCTCCGTGGTACC | CCGG | Endogenous target site of human |
| GRIN2B | G4 | AGAGTAGGCTGGTAGATGGAG | TTGG | Endogenous target site of human |
| GRIN2B | G5 | GTCAGACATGAGATCACAGAT | GCGG | Endogenous target site of human |
| GRIN2B | G6 | TTCATGGCTACCAGTTCCACC | CGGG | Endogenous target site of human |
| GRIN2B | G7 | AGGCTCAAAGGGAAGATACAT | CTGG | Endogenous target site of human |
| GRIN2B | G8 | CATCTAACAAGGGAGAAAGTG | AAGG | Endogenous target site of human |
| GRIN2B | G9 | AAGGCTCATAGTAATCGTCTG | AAGG | Endogenous target site of human |
| GRIN2B | G10 | TGTGAGCCTCAGCTTCCCTGG | TGGG | Endogenous target site of human |
| GRIN2B | G11 | TGGAAGCCTGCTGCAGCCACA | CGGG | Endogenous target site of human |
| GRIN2B | G17 | TTGTACTTAAAAGACATGCTT | TCGG | Endogenous target site of human |
| Site3 | S3 | AAGAATACTAAGCATAGACTC | CAGG | Endogenous target site of human |
| RUNX1 | R13(RUNX1_13) | GAAAGAGAGATGTAGGGCTAG | AGGG | Endogenous target site of human |
| EMX1 | E0(EMX1_1) | ATAGGGTTAGGGGCCCCAGGC | CGGG | Endogenous target site of human for GUIDE-seq |
| EMX1 | E2(EMX1_2) | ACATTCACAGAAGGGGATGGC | AGGG | Endogenous target site of human for GUIDE-seq |
| RUNX1 | R13(RUNX1_13) | GAAAGAGAGATGTAGGGCTAG | AGGG | Endogenous target site of human for GUIDE-seq |
